# Transcranial direct current stimulation with functional magnetic resonance imaging: a detailed validation and operational guide

**DOI:** 10.12688/wellcomeopenres.16679.1

**Published:** 2021-06-07

**Authors:** Davide Nardo, Megan Creasey, Clive Negus, Katerina Pappa, Alphonso Reid, Oliver Josephs, Martina F. Callaghan, Jenny T. Crinion

**Affiliations:** 1 Institute of Cognitive Neuroscience, University College London, London, UK; 2 MRC Cognition and Brain Sciences Unit, University of Cambridge, Cambridge, UK; 3 Wellcome Centre for Human Neuroimaging, University College London, London, UK; 4 Institute of Health and Wellbeing, University of Glasgow, Glasgow, UK

**Keywords:** transcranial direct current stimulation, transcranial electrical brain stimulation, fMRI, functional magnetic resonance imaging, standard operating procedure, safety factors, technical challenges, implementation guide

## Abstract

**Introduction:** Transcranial direct current stimulation (tDCS) is a non-invasive brain stimulation technique used to modulate human brain and behavioural function in both research and clinical interventions. The combination of functional magnetic resonance imaging (fMRI) with tDCS enables researchers to directly test causal contributions of stimulated brain regions, answering questions about the physiology and neural mechanisms underlying behaviour. Despite the promise of the technique, advances have been hampered by technical challenges and methodological variability between studies, confounding comparability/replicability.

**Methods:** Here tDCS-fMRI at 3T was developed for a series of experiments investigating language recovery after stroke. To validate the method, one healthy volunteer completed an fMRI paradigm with three conditions: (i) No-tDCS, (ii) Sham-tDCS, (iii) 2mA Anodal-tDCS. MR data were analysed in SPM12 with region-of-interest (ROI) analyses of the two electrodes and reference sites.

**Results: **Quality assessment indicated no visible signal dropouts or distortions introduced by the tDCS equipment. After modelling scanner drift, motion-related variance, and temporal autocorrelation, we found no field inhomogeneity in functional sensitivity metrics across conditions in grey matter and in the three ROIs.

**Discussion: **Key safety factors and risk mitigation strategies that must be taken into consideration when integrating tDCS into an fMRI environment are outlined. To obtain reliable results, we provide practical solutions to technical challenges and complications of the method. It is hoped that sharing these data and SOP will promote methodological replication in future studies, enhancing the quality of tDCS-fMRI application, and improve the reliability of scientific results in this field.

**Conclusions**: The method and data provided here provide a technically safe, reliable tDCS-fMRI procedure to obtain high quality MR data. The detailed framework of the Standard Operation Procedure SOP ( https://doi.org/10.5281/zenodo.4606564) systematically reports the technical and procedural elements of our tDCS-fMRI approach, which we hope can be adopted and prove useful in future studies.

## Introduction

Transcranial direct current stimulation (tDCS) is one method of non-invasive transcranial electrical brain stimulation (tES). The technique uses a small current (1-2mA) applied via scalp electrodes for up to 20 minutes in human volunteers. During tDCS stimulation current flows between the surface electrodes – passing through the brain to complete a circuit. Increasing interest in the technique has stemmed from a desire to explore and alter the physiological mechanisms underlying basic human motor, perceptual and cognitive processes ( Nitsche *et al*., 2008). Its immediate and long-lasting effects, albeit with unpredictable cognitive results ( Thair *et al*., 2017), its safety and tolerability ( Antal *et al*., 2017; Bikson *et al*., 2016), non-complex technical requirements, and low cost ( Woods *et al*., 2016) have made it an attractive treatment option for several neurological and psychiatric disorders ( Brunoni *et al*., 2019; Schulz *et al*., 2013). However, the neural mechanisms by which tDCS modulates human brain and behavioural function are still unclear. An increased understanding of these mechanisms would allow more effective and individualised targeted interventions to be developed.

With the advent of magnetic resonance imaging (MRI)-compatible tES devices, concurrent tDCS and functional magnetic resonance imaging (fMRI) is technically feasible. Using the “perturb and measure” approach ( Paus, 2005) the casual contributions of a stimulated brain region’s function can be directly assessed online, during (cognitive) task performance, offering researchers a unique opportunity to answer basic questions about underlying physiology. Combined with the high spatial resolution that fMRI offers across the entire brain, research has shown that tDCS effects are not spatially restricted to the brain region directly underneath the stimulating electrode. Indeed, tDCS affects multiple regions due in part to distributed current flow and brain connectivity ( Abellaneda-Perez *et al*., 2020; Mondino *et al*., 2020), including anatomically distant but functionally connected regions ( Chib *et al*., 2013; Stagg *et al*., 2013). This has resulted in a number of important guides published on the technique ( Meinzer *et al*., 2014; Thair *et al*., 2017; Woods *et al*., 2016). A number of additional studies have illustrated that the technique can be conducted safely (e.g., minimising risk of local electrode heating and skin burning) without posing severe data quality constraints as long as proper procedures are followed ( Antal *et al*., 2014; Esmaeilpour *et al*., 2020). Nonetheless, advances in the field of tDCS-fMRI have been hampered by the methodological variability between studies, which limits comparisons between studies and replicability of findings ( Esmaeilpour *et al*., 2020).

Surprisingly, despite an increasing number of research labs using tES and fMRI, a recent systematic review of 222 tES-fMRI experiments (181 tDCS) published before February 1, 2019, found there were no two studies with the same methodological parameters to replicate findings ( Ghobadi-Azbari *et al*., 2020). The authors conclude that, because the methodology progressed largely independently between different research groups, it resulted in diverse protocols and findings across research groups. Importantly, the heterogeneous mixture of findings, cannot always be interpreted independently from the methodological parameters ( Nitsche *et al*., 2015). Indeed, concurrent tES-fMRI studies are more susceptible to artefactual noise than other fMRI scenarios and may risk false positive BOLD (blood oxygen level dependent) signal results ( Antal *et al*., 2014; Woods *et al*., 2016). Very few studies have provided data on change in the magnetic field in relation to concurrent tDCS-fMRI, the magnitude and nature of which are likely to depend on the exact experimental setup within each lab, for each fMRI paradigm. This highlights the need for careful consideration of tDCS-fMRI results and how the lack of methodological overlap between studies to date makes any meta-analysis and/or conclusion about mechanistic effects of tDCS extremely challenging.

To address the inconsistency of methodological approaches used in tDCS-fMRI studies, here we provide a step-by-step guide through the standard operating procedure ( SOP) governing safe operation of tDCS-fMRI at the Wellcome Centre for Human Neuroimaging (WCHN). The SOP was designed to provide sufficiently detailed methodological information so that methods can be precisely replicated ( Callaghan, 2021). It was originally developed for a series of experiments using tDCS-fMRI investigating language recovery after stroke, but can be adapted for any study which uses fMRI to investigate the mechanisms underlying tDCS effects. At the time of writing, we have collected data from over 36 stroke patients with aphasia with no reported adverse events or tolerance issues ( Ondobaka *et al*., 2020), and the same fMRI-tDCS procedure has been found to be well tolerated by healthy older adults ( Holland *et al*., 2011). In both these studies, participants were not able to reliably detect differences between the stimulation conditions (anodal 2mA and sham tDCS).

Here we focus on methods required for the safe use of tDCS equipment in the MRI environment, whilst maintaining high image quality to obtain reliable fMRI results. Detailed investigation of a single subject’s data across stimulation conditions compared to a baseline, No-tDCS condition, validating the approach is followed by a discussion of the key risk factors (safety and image artefact) associated with concurrent tDCS-fMRI, and risk mitigation strategies implemented in our Lab. By sharing this information, we aim to aid replication of methodological approaches across studies and sites and increase replicability of published evidence in the field.

## Methods

### Ethical considerations

Data were acquired with approval from the UCL Research Ethics Committee (8711/001) and with the informed written consent of a healthy participant (female, scientist, 46 years old) recruited within UCL in September 2020 at the WCHN.

### tDCS equipment and procedure

We used MR-compatible tDCS equipment (NeuroConn DC-stimulator) to apply 2mA anodal tDCS delivered for 20 mins to the frontal cortex. In the full Standard Operating Procedure ( SOP), we report Step-by-step the detailed procedure ( Callaghan, 2021) we have put in place. In what follows we report a summary and highlight additional bespoke steps, we took to optimise consistency of our method. Besides MRI safety contraindications such as metallic implants, pacemaker, claustrophobia, the participant was screened for additional tDCS-specific contraindications ( Poreisz *et al*., 2007) including: the presence of severe or frequent migraines, the presence of metal implants, the use of any stimulators or implants, the presence of epilepsy, or the presence of serious or recent head trauma, because neurochemical changes may modify the flow of the current ( Datta *et al*., 2010). tDCS equipment was set-up in three different environments as per equipment safety guidelines: 1) Testing room - for the intial tDCS electrode tDCS-specific contraindications ( DaSilva *et al*., 2011). and participant paradigm set-up, to ensure participant was happy with stimulation sensation ( Fertonani *et al*., 2015) and safe to proceed to MR environment, 2) MR Control room - where the tDCS non-MR compatible stimulator components reside, 3) Scan room – tDCS MR compatible electrodes and stimulator components. Participants were suitably prepared in the Testing room before coming to the scanner, including MR-safety checks and tDCS impedance checks. Within the Testing room, EEG conductive paste (Ten20) was used as the electrode contact medium, and 3M Coban elastic wrap bandage was used to secure electrode placement. In the MR Control room, the tDCS stimulator was placed inside a tailormade radiofrequency (RF) shielded box during the experiment, to minimise any RF interference between the Scan Room and the external environment, and tDCS stimulation was initiated by the scanner via a Fibre-Optic trigger. In the Scan room a tailor-made foam-base was used to facilitate equipment and cable set-up in the scanner bore, and guarantee placement consistency across sessions. The participant was connected to the tDCS equipment by making sure that no loops were created in the scanner, and loops were avoided along the whole length of the various cables in the Scan Room. A detailed procedure to safely remove participants from the scanner/tDCS equipment in case of emergency is also provided in our full SOP ( Callaghan, 2021). Our procedure involved three people (typically two researchers and one radiographer), whose roles and responsibilities are defined in detail.

### Quality assessment of MR images

In order to compare the functional sensitivity of the three stimulation conditions (i.e., No-tDCS setup in place, Sham-tDCS, and Anodal-tDCS), we computed the t-score testing for the mean signal (cf. Corbin *et al*., 2018) using Statistical Parametric Mapping software (SPM12) running under Matlab 2020a (MathWorks, Natick, MA). Matlab scripts for this project can be accessed here ( Callaghan, 2021). Matlab scripts are largely compatible with and may be run in the open source alternative GNU Octave. In the simple case of the design matrix being a unitary vector, with length corresponding to the number of temporal samples, and there being no temporal correlation in the data, this metric reduces to the commonly used temporal signal-to-noise ratio (tSNR) weighted by the square root of the number of samples. We computed this functional sensitivity metric both at the whole-brain level, restricted to grey matter tissue, and within three regions-of-interest (ROIs) located beneath the anodal electrode, the cathodal electrode, and an independent site remote from the electrodes, which was used as a reference.

### fMRI acquisition

MR data were collected on a 3T Siemens PrismaFit system (Siemens, Erlangen, Germany) at the WCHN. Data included a T1-weighted MPRAGE acquisition for anatomical reference (TR = 2.53 s, TE = 3.34 ms, voxel size = 1 × 1 × 1 mm, field of view = 256 × 256 × 176 mm ^3^), dual gradient-echo based field maps to map B _0_ field inhomogeneity and subsequently apply distortion correction to the functional images, and T2*-weighted echo-planar images (EPI) using a 20-channel head coil and acquired during resting-state, with the following parameters: TR = 3.36 s, TE = 30 ms, 48 axial slices with ascending slice ordering, slice thickness = 2.5 mm, inter-slice gap = 0.5 mm, in-plane resolution = 3 × 3 mm, flip-angle 90°. A total of 70 volumes (65 of interest and 5 dummies) were acquired in each of three consecutive runs, lasting approximately 4 min each.

### fMRI preprocessing

Functional data were preprocessed and analysed in native space as defined by (i.e., co-registered with) the anatomical image using Statistical Parametric Mapping software (SPM12) running under Matlab 2020a (MathWorks, Natick, MA). All functional volumes of interest were realigned and unwarped using session-specific voxel displacement maps derived from the B _0_ mapping data. The structural image was segmented into grey matter, white matter, and cerebrospinal fluid.

### fMRI analysis

Statistical analyses were performed in a run-specific fashion. Parameter estimates were calculated for all brain voxels using the General Linear Model (GLM) in SPM12. To remove any low-frequency scanner drifts, data were high-pass filtered using a set of discrete cosine functions with a cut-off period of 128s. A GLM consisting of the nuisance regressors describing motion-related variance (given the experiment was task-free) and temporal autocorrelation was evaluated, and the t-score testing for the mean signal extracted.

### Regions-of-interest (ROIs)

**Figure 1.**
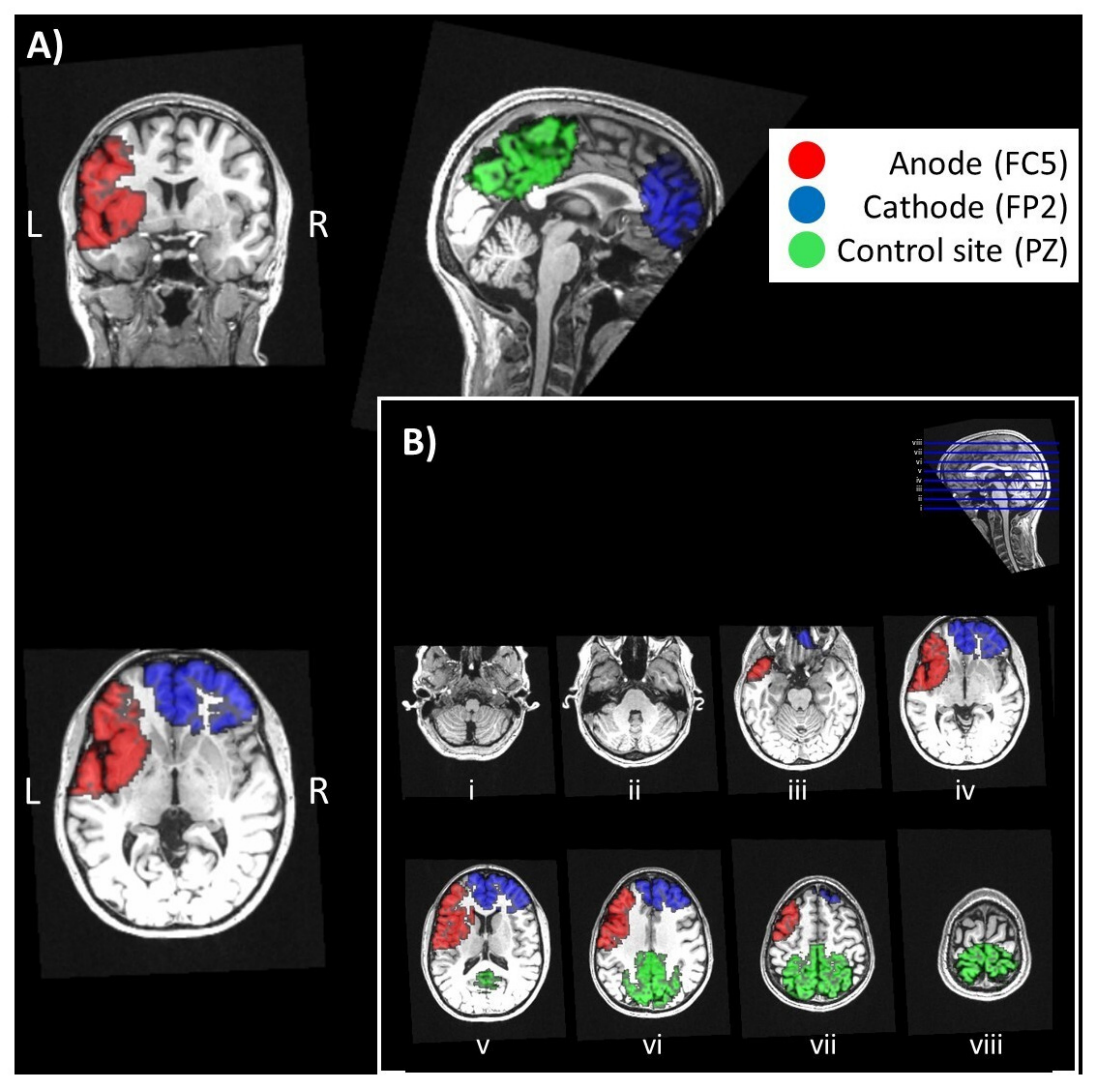
Regions-of-interest (ROIs). **A**) Locations of the three ROIs tested overlaid on the coronal, sagittal, and axial sections of a T1-weighted image. ROIs were defined beneath the anodal electrode (in red, corresponding to FC5 in a 10-20 system), cathodal electrode (in blue, corresponding to FP2) and at an independent site remote from the electrodes, used as a reference (in green, corresponding to PZ). **B**) Locations of the three ROIs tested overlaid on the axial sections shown in Figure 2.

To test more specifically for any local changes within the vicinity of the stimulating electrodes, a series of regions of interest (ROIs) were created in two steps. First, spheres with a 40mm radius were created around the cortical projections (using the anatomical image as a reference) of the anode electrode (corresponding to FC5 in a 10-20 system), cathode electrode (corresponding to FP2), and an independent site remote from both electrodes as a reference (corresponding to PZ), using the MarsBaR toolbox for SPM. Second, each of these spherical ROIs (in native space) was (inclusively) combined with the whole-brain segmented grey matter tissue from the T1-weighted image. Figure 1 illustrates the locations of the three ROIs tested overlaid on axial brain sections.

## Results

**Figure 2.**
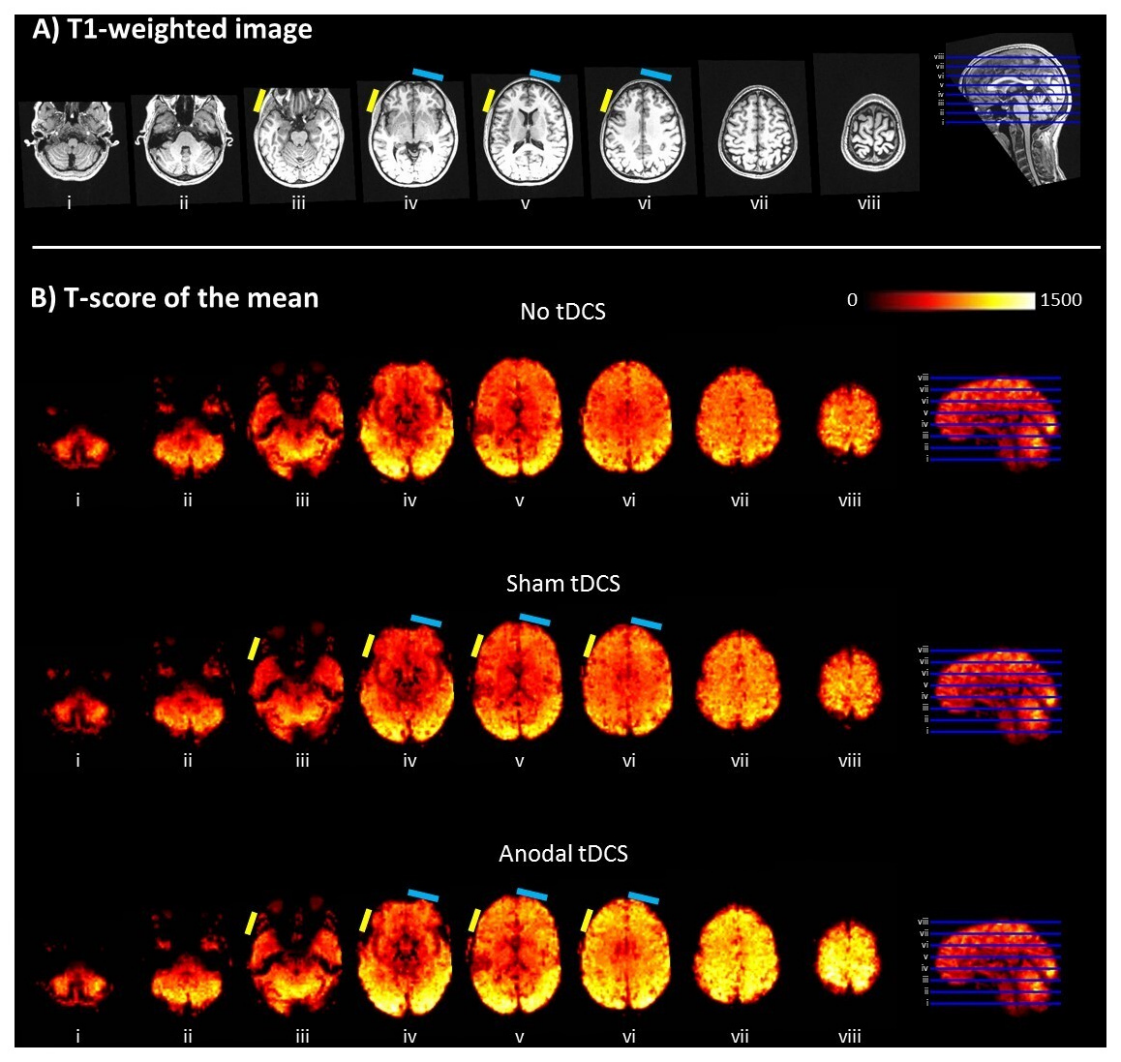
Quality assessment of MR images acquired on one participant. **A**) High-resolution (1mm, isotropic) structural T1-weighted image denoting electrode locations shown as a reference. **B**) t-score of the mean maps. In all panels, axial slices (in ascending order from *i* to *viii* with location denoted by the blue lines on the sagittal section on the right) are 15mm apart. The approximate position of the electrodes is indicated by the coloured small rectangles (anodal = yellow; cathodal = light blue). There are no visible signal dropouts or distortions introduced by the electrodes and/or conductive paste. The functional sensitivity measures (t-scores of the mean) are comparable across stimulating conditions, though highest in the Anodal-tDCS case.

Quality assessment of whole brain MR images indicated no visible signal dropouts or image distortions introduced by the tDCS electrodes, equipment and/or conductive paste ( Callaghan, 2021). This was in both a high-resolution (1mm, isotropic) structural T1-weighted image and EPI images across the three conditions (No-tDCS, Sham, and Anodal stimulation). While functional sensitivity measures (t-scores of the mean EPI maps) were broadly comparable at a whole-brain level across the three conditions. This indicates that functional MR sensitivity was not degraded or adversely affected by the tDCS set-up and stimulation protocol. These results are displayed in Figure 2.

**Figure 3.**
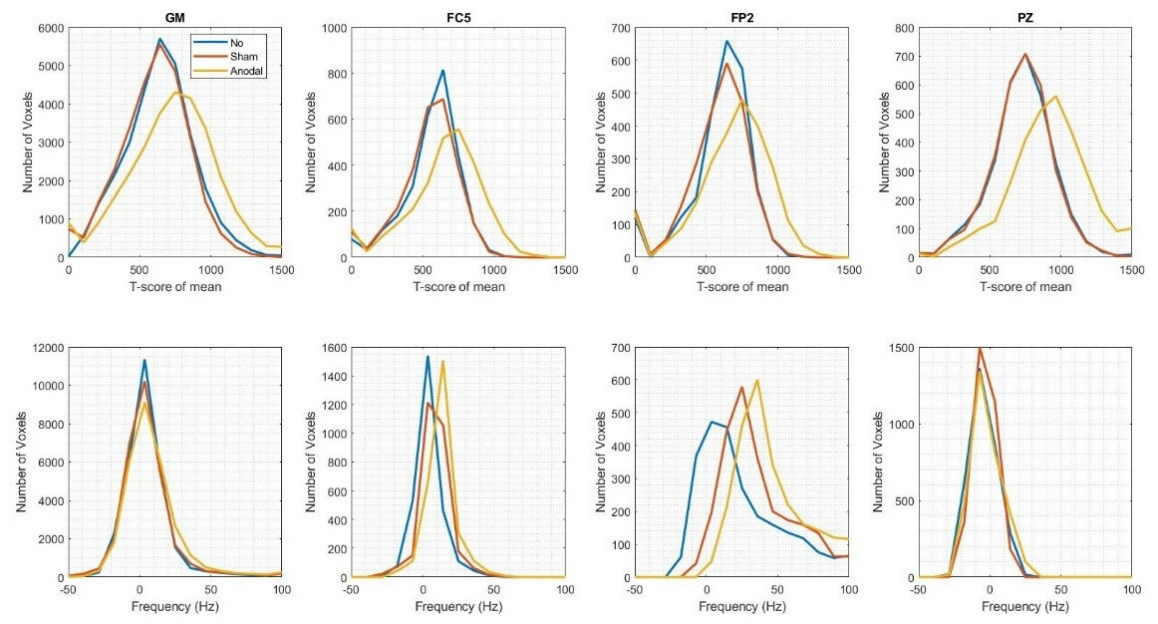
Functional sensitivity metrics across conditions in grey matter (GM), (leftmost column) and in the three regions of interest (ROIs) (from left to right: anodal electrode – FC5, cathodal electrode – FP2, independent site remote from the electrodes – PZ). **Top row:** t-score of the mean. This measure is extracted from the GLM used for the functional analyses after modelling scanner drift, motion-related variance, and temporal autocorrelation. The t-scores of the mean values with the tDCS equipment in place (i.e., Anodal and Sham conditions) are comparable, if not higher than the No-tDCS condition (i.e., functional sensitivity was not degraded). **Bottom row:** Offset frequency (measured in Hz) to capture field inhomogeneity. The substantial overlap among the three distributions shows that no field inhomogeneity is introduced by the tDCS equipment. Global offsets are observed between conditions in the cathodal (FP2) ROI, but the distribution is in fact broader, indicative of poorer field homogeneity, in the No-tDCS condition.

Region-Of-Interest (ROI) analyses investigated the frequency distribution and t-score of the mean values extracted from the grey matter (GM) anode (FC5), cathode (FP2) and reference (PZ) ROIs. The results illustrated in Figure 3 found there was a high level of overlap for the t-score of the mean distribution values in the Sham- and No-tDCS conditions extracted from each of the ROIs. A shift to higher t-score of the mean values was evident for the Anodal-tDCS case. The width of frequency distributions in each ROI reflecting field inhomogeneity, was not increased across conditions, indicating that no field inhomogeneity was introduced by the tDCS equipment or stimulation condition. A frequency distributions overlap was observed in each ROI except the cathode ROI (FP2), where a shift to higher frequency was observed. Here global offsets were observed between conditions, but the distribution was in fact broader, indicative of poorer field homogeneity, in the No-tDCS condition.

In summary, taken together these whole-brain and ROI results indicate functional sensitivity was not degraded and no field inhomogeneity was introduced by the tDCS equipment and stimulation condition.

## Discussion

Combining non-invasive neuro-stimulation and functional neuroimaging techniques can provide a unique opportunity to understand the immediate and long-lasting effects of transcranial direct current stimulation (tDCS) on the brain. At the WCHN, tDCS is being used alongside functional magnetic resonance imaging (fMRI) in order to understand the neural mechanisms underlying tDCS behavioural effects. The combination of fMRI and tDCS methods, including simultaneous/concurrent tDCS-fMRI application can provide unique insight into the neuromodulatory effects of tDCS not only in the targeted brain regions, but also their interconnected networks. The ultimate aim of these mechanistic experiments is to find a relationship between behavioural and neural responses to tDCS. Here, we have shared our detailed procedural methodologies with the aim of increasing replicability of tDCS-fMRI methods and reliability of results of future studies. We hope this will in turn enable the field to gain greater insight into the mechanisms underpinning neural and behavioural modulation by tDCS, which would open up new directions within scientific research and clinical applications, such as developing targeted and meaningful therapies.

In this section, we first focus on discussing the identified safety risks and accompanying risk mitigations that are specific to the incorporation of the tDCS equipment into the MRI environment that were considered critical during the writing of this operational procedure. Then we discuss the specific challenges of concurrent tDCS-fMRI data and how acquisition of appropriate B _0_ field map data can help allay concerns over artefacts and false positive functional results from perturbation of the magnetic field. To date, this protocol has proved a safe and reliable means of obtaining high quality fMRI data concurrently with the application of 2mA anodal tDCS (20 mins) in over 18 healthy older adults and 36 aphasic stroke patients.

### Safety considerations for tDCS in the MRI environment

The MRI environment poses a number of significant risk factors, primarily due to the various comparatively strong magnetic fields used, which can vary in both space and time. The main magnetic field (3 Tesla in our case) can exert significant forces on ferrous objects or current-carrying conductors. The primary risk to be mitigated against is the introduction of any ferrous components. The NeuroConn DC stimulator Outer-Box contains an RF filter with ferrous components. This Outer-Box should never enter the Scan Room to prevent it from becoming a projectile under the force exerted by the main magnetic field. It should be housed within the waveguide and always be placed and removed via the Control Room.

The time-varying magnetic fields used to achieve the excitation process in MRI have associated electric fields (i.e., this is an electromagnetic RF field). These can induce current flow, both in the participant and any equipment, and lead to heating. During scanning, the MRI system continually monitors the transmitted power to ensure it is as expected, and within regulatory limits by modelling the specific absorption rate, i.e., the energy deposition in the participant, in the absence of any equipment. To accommodate the introduction of the tDCS equipment, and ensure it does not invalidate the model, further mitigation strategies are adopted. Care is taken to arrange cables without introducing any closed loops in which current could flow, and the electrode leads are run along the centre of the bore using the bespoke Foam-base. This base has a groove that maximises the safety of the cabling configuration by ensuring it is parallel with the bore, centred within the transmitting RF coil’s volume without any loops, and running away from the participant. The tDCS equipment also has multiple RF filters incorporated, and a high input impedance to minimise the currents that could flow as a result of any induced voltage, which could also be caused by the rapidly switching imaging gradients. Resistors are incorporated into the leads adjacent to the electrode pads to further limit any possible current flow. The MRI compatible electrodes are made from an electrically conductive rubber. It is possible that circumferential RF currents could be set up directly within these relatively large pads but for the low SAR sequences used heating is negligible, as confirmed by previous experiments ( Holland *et al*., 2011). As a further risk mitigation strategy, in our Lab only low power imaging sequences are used.

The MRI scanner is situated within a Faraday cage, a continuous copper foil on a wooden support structure, designed to be impermeable to RF fields and often referred to as the RF cage. The purpose of this cage is to contain any internally-generated RF sources, e.g. the transmit coil, within the Scan Room and to prevent any external sources from the everyday environment, which could reduce image quality, from entering the Scan Room. The RF cage is explicitly grounded at a single point to prevent unintended connections to the building’s electrical ground. To preserve this condition, all electrical connections to the cage are made via a so called “penetration panel” and are RF filtered to maintain the intended RF isolation. Most RF filters used for this purpose are formed of capacitors and inductors in which the capacitors are connected to the RF screen of the cage, which is itself connected to ground. This means that the filters provide a pathway to ground. If any equipment entering the Scan Room in this way is connected to the participant, they too become part of a grounded circuit. The tDCS equipment has been designed for stimulation of human participants in the MRI environment according to the International Electrotechnical Commission standards (60601-1 Class 1 [battery powered] with Type BF [Body, Floating, i.e., no possible route to ground] applied part standard). The NeuroConn manufacturer achieves this by using electrically insulated non-grounded filters, which ensure that the participant being scanned is not connected to ground. This is an intrinsically safe arrangement because, even if a fault condition were to develop during scanning, such that the participant was brought into contact with high voltages, no conducting path is available for a dangerous current to flow through the participant to ground via the tDCS apparatus. To maintain this BF safety status, the penetration panel has not been used to integrate the tDCS equipment in our lab.

The other means of penetrating the RF cage is via a waveguide – a long cylindrical tube that will only allow signals above a certain frequency, known as the cut-off frequency, to pass and therefore can be used to exclude RF signals that would otherwise interfere with imaging. This is the approach we have used to integrate the tDCS equipment into the scanner environment. While this approach ensures that the equipment and participant are not connected to ground, it also introduces the risk of violating the RF isolation (a data quality requirement) and allowing RF from the external environment into the Scan Room.

Two filters are used to attenuate current flow: the MR-compatible “Inner-Box” minimises any currents flowing in the section of the electrode lead in the bore, while the incompatible “Outer-Box” in the waveguide itself prevents current flow in the outer cabling entering the Scan Room. However, these RF filters have limited performance meaning that a risk of RF interference compromising the imaging remains, particularly if there is an equipment fault. This risk motivated the extension of our RF cage to enclose the entire stimulator within a shielded box attached, via shielded flexible metallic tubing, to the outside of the waveguide. This additional box has a removable shielding lid, which creates a robust RF seal via fingerstrip gaskets (e.g., https://hollandshielding.com/Shielding-gasket-solutions-materials#Fingerstrips) and a viewing aperture that is small enough not to compromise the RF shield but sufficient for operation and monitoring of the tDCS device. The lid also proves a useful means of ensuring that the researcher can remain blind to the experimental tDCS condition.

The electrical signal used by the trigger input to drive the stimulator is galvanically isolated from the rest of the circuitry by the manufacturer (cf. manual). When the box is manually triggered, this isolation can be verified by visual inspection since no wires are connected to anything that could be grounded. In theory, an electrical cable can normally be connected directly from the controlling computer, as long as the galvanic isolation is certain. However, in our case such a connection would also have compromised the additional RF screening by providing a path for RF current to flow. Therefore, a fibre-optic trigger signal from the Stimulus PC enters the shielded box through a small waveguide and is subsequently converted to an electrical signal, via battery-control, to drive the stimulator.

### tDCS and fMRI image quality and safety control study

Prior to any neuroscience experiments, and in particular due to the extension to the RF cage, the tDCS equipment setup should be tested to ensure that the integrity of the RF cage isolation had not been compromised. In our case, this was done by measuring the cross-talk between two adjacent MRI scanners with and without the tDCS equipment in place. These tests confirmed that, with the RF shielding solution employed here, the RF noise level was equivalent regardless of the presence of the tDCS equipment. As with any experimental setup, routine quality assurance should also be employed. All equipment should be regularly inspected for damage and maintained in keeping with manufacturer guidelines.

The introduction of an electrical current into the scanner’s magnetic field results in further warping of the magnetic field (i.e., field artefact). This artefact is of critical concern for BOLD fMRI protocols, as it may result in false positive patterns in BOLD signal ( Antal *et al*., 2014; Woods *et al*., 2016). Online tES-fMRI studies are therefore more susceptible to artefactual noise than other fMRI scenarios, the magnitude and nature of which are likely to depend on the exact experimental setup within each Lab, for each experiment, across participants. This highlights the importance of having a replicable set-up with properly placed and shielded electrode cables and stimulation equipment within the scanning area. For example, one study demonstrated evidence of BOLD signal within brains of two cadavers during a concurrent tDCS and fMRI protocol ( Antal *et al*., 2014). Whilst a previous study from our lab demonstrated visual evidence of change in echo-planar imaging (EPI) field maps that was limited to the scalp/surface near to the electrode site ( Holland *et al*., 2011). These contrasting cases demonstrate the need for careful consideration of concurrent tDCS-fMRI data, and acquisition of appropriate field map data to allay concerns over false positive functional results from perturbation of the magnetic field.

**Figure 4.**
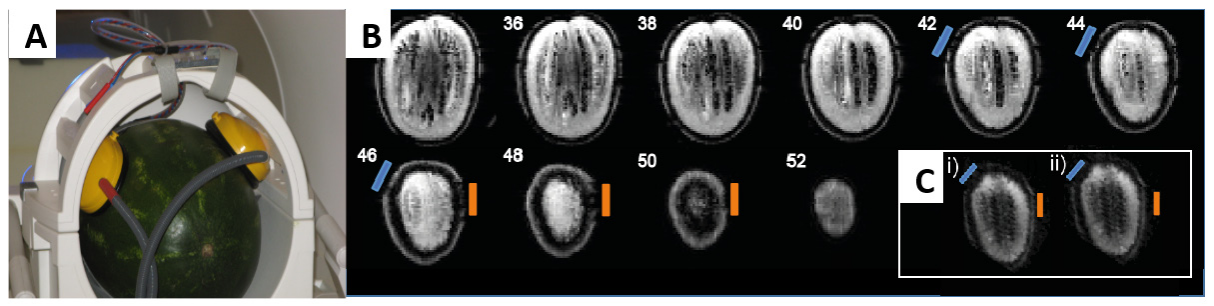
Control concurrent tDCS and fMRI study. ( **A**) A watermelon of similar size to a human adult head was chosen as a continuous 2mA anodal DC could be passed through the surface. The headphones and tDCS electrodes were positioned on the object in the same orientation, and with the same tDCS/fMRI set-up as was used for the main human study (cf. Holland *et al*., 2011); ( **B**) Multi-slice coronal view of the watermelon field distortion. The blue bar indicates the location of the anode electrode, orange the cathode electrode where mild perturbation of the signal is evident in slices 42-50 at the surface layer of the watermelon under each electrode; ( **C**) EPI from slice 46 (watermelon data) for i) un-stimulated (sham) and ii) stimulated (A-tDCS) fMRI runs. This figure has been used with permission from Holland *et al*., 2011.

To address this, prior to scanning human participants, we recommend a control tDCS concurrent with fMRI study for evaluation of the set-up in each Lab. The purpose of the control study is twofold: (i) to ensure the safety of concurrent tDCS and fMRI, and (ii) to quantify any noise effects in the images induced by tDCS delivered simultaneously with the task stimuli. For example, in a previous experiment we delivered 2mA anodal tDCS stimulation for 20 minutes concurrently with the identical stimulus delivery set-up as used in the main study to an inert object (a watermelon) ( Holland *et al*., 2011). Results indicated that: (i) during stimulation no significant changes in surface temperature were detected over time; and (ii) in distortion correction field maps only minimal perturbation of signal was observed at the electrode site (see Figure 4).

The signal distortion was restricted to the surface of the watermelon only. Processing of the acquired functional data found no effects of tDCS on sham or stimulated runs. Together, these results indicated no imaging artefacts induced by the tDCS/fMRI set-up that could account for the effects of A-tDCS reported in the subsequent human experimental study (cf. Holland *et al*., 2011 for full details of effects of tDCS on EPI data). A control study such as this, comparing fMRI data for a short duration of time under two tDCS conditions, e.g., anodal vs sham, can be adapted for any experimental paradigm and tDCS-fMRI set-up.

In the human validation study presented here, the variability introduced by the tDCS equipment appears to be far below the level of variability arising from participant repositioning, reaffirming the importance of careful positioning. Generally higher t-score of the mean values were observed for the Anodal-tDCS case (cf. Figure 1 and Figure 3). However, given that we would not expect an increase in functional sensitivity when using tDCS, this is more likely to originate from variability in participant repositioning, and scanner adjustments, across the different experimental conditions. Indeed, in the Anodal-tDCS case, the participant was positioned furthest into (superiorly) the sensitive volume of the receiving coil, boosting sensitivity. The cathode ROI (FP2) had greatest field inhomogeneity regardless of imaging condition. A frequency offset was observed in this ROI for the Anodal-tDCS case, however, there was no broadening of the frequency distribution. This indicates that the introduction of the tDCS equipment did not increase the field inhomogeneity. In fact, the broadest distribution was observed in this ROI for the No-tDCS condition.

## Conclusion

In this paper, we deliver the Wellcome Centre for Human Neuroimaging standard operating protocol ( SOP) for technically sound and safe application of tDCS concurrently with fMRI. Although the MR-compatible tDCS technique is seemingly simple and easy to apply, we discuss specific aspects that must be taken into consideration when integrating the approach into an MRI environment to obtain both a safe experimental set-up and reliable results. This SOP and the experimental data validating its efficacy is provided as a detailed framework to systematically report the main technical elements of tDCS-fMRI, which can be adopted and used as a baseline for prospective real-world applicability. It is hoped that this will enhance the quality of tDCS-fMRI application in future studies, help provide practical solutions to the technical challenges and complications of the method, and therefore improve the quality of scientific work in this field further.

## Data availability

### Underlying data

Zenodo: WCHN/tDCS_fMRI: tDCS for fMRI SOP Release. https://doi.org/10.5281/zenodo.4606564 Callaghan (2021).

This project contains the following underlying data:

- Andodal_tDCS
- MPRAGE
- No_tDCS
- Sham_tDCS

### Extended data

Zenodo: WCHN/tDCS_fMRI: tDCS for fMRI SOP Release. https://doi.org/10.5281/zenodo.4606564 Callaghan (2021).

This project contains the following underlying data:

- WCHN_tDCS_fMRI_SOP.pdf (Standard operating procedures)

## Software availability

- Source code available from: https://github.com/WCHN/tDCS_fMRI/tree/v1.0.0
- Archived source code at time of publication: https://doi.org/10.5281/zenodo.4606564 Callaghan (2021).
- License: GNU General Public License v3.0
