# Transcranial direct current stimulation with functional magnetic resonance imaging: a detailed validation and operational guide

**DOI:** 10.12688/wellcomeopenres.16679.2

**Published:** 2023-02-06

**Authors:** Davide Nardo, Megan Creasey, Clive Negus, Katerina Pappa, Ali Aghaeifar, Alphonso Reid, Oliver Josephs, Martina F. Callaghan, Jenny T. Crinion

**Affiliations:** 1 MRC Cognition and Brain Sciences Unit, University of Cambridge, Cambridge, UK; 2 Department of Education, University of Roma Tre, Rome, Italy; 3 Wellcome Centre for Human Neuroimaging, University College London, London, UK; 4 Institute of Cognitive Neuroscience, University College London, London, UK; 5 Institute of Health and Wellbeing, University of Glasgow, Glasgow, UK

**Keywords:** transcranial direct current stimulation, transcranial electrical brain stimulation, fMRI, functional magnetic resonance imaging, standard operating procedure, safety factors, technical challenges, implementation guide

## Abstract

**Introduction**: Transcranial direct current stimulation (tDCS) is a non-invasive brain stimulation technique used to modulate human brain and behavioural function in both research and clinical interventions. The combination of functional magnetic resonance imaging (fMRI) with tDCS enables researchers to directly test causal contributions of stimulated brain regions, answering questions about the physiology and neural mechanisms underlying behaviour. Despite the promise of the technique, advances have been hampered by technical challenges and methodological variability between studies, confounding comparability/replicability.

**Methods**: Here tDCS-fMRI at 3T was developed for a series of experiments investigating language recovery after stroke. To validate the method, one healthy volunteer completed an fMRI paradigm with three conditions: No-tDCS, Sham-tDCS, Anodal-tDCS. MR data were analysed with region-of-interest (ROI) analyses of the electrodes and reference site.

**Results**: Quality assessment indicated no visible signal dropouts or distortions in the brain introduced by the tDCS equipment. After modelling scanner drift, motion-related variance, and temporal autocorrelation, we found that functional MR sensitivity was not degraded or adversely affected by the tDCS set-up and stimulation protocol across conditions in grey matter and in the three ROIs.

**Discussion**: Key safety factors and risk mitigation strategies that must be taken into consideration when integrating tDCS into an fMRI environment are outlined. To obtain reliable results, we provide practical solutions to technical challenges and complications of the method. It is hoped that sharing these data and Standard Operation Procedure (SOP) will promote methodological replication in future studies, enhancing the quality of tDCS-fMRI application, and improve the reliability of scientific results in this field.

**Conclusions**: Our method and data provide a technically safe, reliable tDCS-fMRI procedure to obtain high quality MR data. The detailed framework of the SOP systematically reports the technical and procedural elements of our tDCS-fMRI approach, which can be adopted and prove useful in future studies.

## Introduction

Transcranial direct current stimulation (tDCS) is one of several methods of non-invasive transcranial electrical brain stimulation (tES). There is a great deal of variability of protocols that can be used depending on the research questions at hand, but with healthy volunteers these will typically use a small current (1-2 mA) applied via scalp electrodes for up to 20 minutes in human volunteers, although a longer duration of up to 40 minutes has been adopted more recently ( Pinto *et al.*, 2018). During tDCS stimulation, current flows between the surface electrodes – passing through the brain to complete a circuit. Increasing interest in the technique has stemmed from a desire to explore and alter the physiological mechanisms underlying basic human motor, perceptual and cognitive processes ( Nitsche *et al*., 2008). Its immediate and long-lasting effects, albeit with unpredictable cognitive results ( Thair *et al*., 2017), its safety and tolerability ( Antal *et al*., 2017; Bikson *et al*., 2016), non-complex technical requirements, and low cost ( Woods *et al*., 2016) have made it an attractive treatment option for several neurological and psychiatric disorders ( Brunoni *et al*., 2019; Schulz *et al*., 2013). However, the neural mechanisms by which tDCS modulates human brain and behavioural function are still unclear. An increased understanding of these mechanisms would allow more effective and individualised targeted interventions to be developed.

With the advent of magnetic resonance imaging (MRI)-compatible tES devices, concurrent tDCS and functional magnetic resonance imaging (fMRI) is technically feasible. Using the “perturb and measure” approach ( Paus, 2005) the casual contributions of a stimulated brain region’s function can be directly assessed online, during (cognitive) task performance, offering researchers a unique opportunity to answer basic questions about underlying physiology. Combined with the high spatial resolution that fMRI offers across the entire brain, research has shown that tDCS effects are not spatially restricted to the brain region directly underneath the stimulating electrode. Indeed, tDCS affects multiple regions due in part to distributed current flow and brain connectivity ( Abellaneda-Perez *et al*., 2020; Mondino *et al*., 2020), which can include anatomically distant, but functionally connected, regions ( Chib *et al*., 2013; Stagg *et al*., 2013). This has resulted in a number of important guides published on the technique ( Meinzer *et al*., 2014; Thair *et al*., 2017; Woods *et al*., 2016). A number of additional studies have illustrated that the technique can be conducted safely (e.g., minimising risk of local electrode heating and skin burning) without posing severe data quality constraints as long as proper procedures are followed ( Antal *et al*., 2014; Esmaeilpour *et al*., 2020). Given the broad range of stimulation procedures that can be used and research questions that can be asked, it is imperative that any methodological variability be minimised so that the biological and task-relevant variance can be isolated and better understood. However, methodological variability between studies has limited the capacity to compare studies and replicate findings ( Esmaeilpour *et al*., 2020); although a recent consensus guide has started to pave the way to resolving this issue ( Ekhtiari *et al.*, 2022).

Surprisingly, despite an increasing number of research Labs using tES and fMRI, a recent systematic review of 222 tES-fMRI experiments (181 tDCS) published before February 1, 2019, found there were no two studies with the same methodological parameters to replicate findings ( Ghobadi-Azbari *et al*., 2020). The authors concluded that, because the methodology progressed largely independently between different research groups, it contributed to diverse protocols and findings across research groups. Importantly, the heterogeneous mixture of findings, cannot always be interpreted independently from the methodological parameters ( Nitsche *et al*., 2015). Indeed, concurrent tES-fMRI studies are more susceptible to artefactual noise than other fMRI scenarios and may risk false positive BOLD (blood oxygen level dependent) signal results ( Antal *et al*., 2014; Woods *et al*., 2016). Very few studies have provided data on change in the magnetic fields or functional sensitivity in relation to concurrent tDCS-fMRI, the magnitude and nature of which are likely to depend on the exact experimental setup within each Lab, for each fMRI paradigm. This highlights the need for careful consideration of tDCS-fMRI results and how the lack of methodological overlap between studies to date (but see Ekhtiari *et al.*, 2022) makes any meta-analysis and/or conclusion about mechanistic effects of tDCS extremely challenging.

In sum, tES studies to date have involved a diversity of research questions, populations tested, and theories of underlying neurophysiological mechanisms, in conjunction with variability in methodologies and parameters adopted. We therefore sought to provide an operational guide that illustrates, in detail, the various procedural steps involved, and mitigation strategies that can be adopted, in order to minimise (or even rule out) the likelihood that differences in methodological implementation drive variability in findings between studies. This is provided in the form of a step-by-step standard operating procedure ( SOP; Nardo *et al.*, 2021a; https://doi.org/10.5281/zenodo.7569833) that governs safe operation of tDCS-fMRI at the Wellcome Centre for Human Neuroimaging (WCHN). The SOP was designed to provide sufficiently detailed methodological information so that publicly available code can be used to replicate the findings reported here. The SOP was developed for a series of concurrent tDCS-fMRI experiments using conventional tDCS configurations and frontal montages. In principle, this can be adapted for any study which uses fMRI to investigate the mechanisms underlying tDCS effects (cf. Discussion). At the time of writing, we have collected high quality data from over 36 stroke patients with aphasia with no reported adverse events or tolerance issues ( Ondobaka *et al*., 2020). The same fMRI-tDCS procedure has also been well tolerated by healthy older adults ( Holland *et al*., 2011; Nardo *et al.*, 2021b). In both studies, participants were unable to reliably detect differences between the stimulation conditions (i.e., anodal 2 mA and sham tDCS).

Here we focus on methods required for the safe use of tDCS equipment in the MRI environment, whilst maintaining high image quality to obtain reliable fMRI results. Detailed investigation of a single subject’s data across stimulation conditions compared to a baseline, No-tDCS condition, is used to illustrate the validity of the approach. This is followed by a discussion of the key risk factors (safety and image artefact) associated with concurrent tDCS-fMRI, and the risk mitigation strategies we have implemented. By sharing this information, we aim to aid replication of methodological approaches across studies and sites and by consequence increase replicability of published evidence in the field.

## Methods

### Ethical considerations

Data were acquired with approval from the UCL Research Ethics Committee (8711/001) and with the informed written consent of a healthy participant (female, scientist, 46 years old) recruited within UCL in September 2020 at the WCHN.

### tDCS equipment and procedure

We used MR-compatible tDCS equipment (NeuroConn DC-stimulator) to apply 2 mA anodal tDCS delivered for 20 minutes to the frontal cortex using rectangular rubber electrodes (5×7 cm), allowing for a current density of 0.057 mA/cm2 ( Holland *et al.*, 2011). The anode was placed over the left inferior frontal cortex (in correspondence with position FC5 in the 10-20 EEG system), whereas the cathode was placed over the contralateral frontopolar cortex (corresponding to FP2). For anodal stimulation, the current was increased slowly (ramp-up phase) during the first 15 seconds to the desired stimulation threshold (2 mA), then a constant direct current (2 mA) was delivered for 20 minutes. At the end of the stimulation period, the current was decreased to 0 mA over 1 second (ramp-down). For sham stimulation, the ramp-up phase was followed by 15 second of 2 mA stimulation, which was immediately followed by a 1 second ramp-down phase. The stimulator works with a pre-defined impedance limit ≤10 ohm, above which the device does not operate. We ensured that the impedance was as small as possible in both anodal and sham stimulation conditions. In the full Standard Operating Procedure ( SOP; Nardo *et al.*, 2021a; https://doi.org/10.5281/zenodo.7569833), we report the detailed procedure step by step. In what follows we only report a brief summary, highlighting key steps we took to optimise our method. In addition to screening for any MRI contraindications, such as metallic implants, a pacemaker or claustrophobia, the participant was screened for additional tDCS-specific contraindications ( Poreisz *et al*., 2007) including: the use of any stimulators or implants, or history of severe or frequent migraines, epilepsy or head trauma because neurochemical changes may modify the flow of the current ( Datta *et al*., 2010). tDCS equipment was set-up in three different environments as per equipment safety guidelines: 1) Testing room – for the initial tDCS electrode setup and testing ( DaSilva *et al*., 2011), to ensure the participant could tolerate the stimulation sensation ( Fertonani *et al*., 2015) and was happy to proceed to the MR environment; 2) MR Control room – where the non-MR compatible tDCS stimulator components reside; 3) Scan room – where the MR compatible tDCS electrodes and stimulator components were used. Before coming to the scanner, participants underwent MR-safety checks and tDCS impedance checks in the Testing room. EEG conductive paste (Ten20) was used as the electrode contact medium, and 3M Coban elastic wrap bandage was used to secure the electrodes in place. In the MR Control room, the tDCS stimulator was placed inside a tailor-made radiofrequency (RF) shielded box during the experiment, to minimise any RF interference between the Scan Room and the external environment. tDCS stimulation was initiated by the scanner via a Fibre-Optic trigger. In the Scan room a tailor-made foam base was used to facilitate equipment and cable set-up in the scanner bore, and guarantee consistent placement of the tDCS cabling across sessions. The participant was connected to the tDCS equipment ensuring that no loops were created in the scanner, or at any other point along the length of the various tDCS cabling in the Scan Room. A detailed procedure to safely remove participants from the scanner and tDCS equipment in case of emergency is also provided in our full SOP ( Nardo *et al.*, 2021a). Our procedure involved three people (typically two researchers and one radiographer), whose roles and responsibilities are defined in detail.

### Quality assessment of MR images

In MRI, the precessional frequency is directly proportional to the magnetic field. We therefore mapped the deviation of the frequency from the expected resonance condition at 3T to examine whether or not the inhomogeneity of the magnetic field is increased by the introduction of the tDCS equipment. 

The amplitude of the MRI signal relative to fluctuations that occur over time is typically characterised by the temporal signal-to-noise ratio (tSNR). The t-score of the mean is a similar measure that can be obtained directly from a general linear model (GLM) analysis. This measure additionally accounts for factors such as temporal auto-correlation, scanner drift, and motion-related variance and has previously been shown to be a better proxy for functional sensitivity than tSNR (cf. Corbin *et al.*, 2018). In the simple case of the design matrix being a unitary vector, with length corresponding to the number of temporal samples, and there being no temporal correlation in the data, this metric reduces to the commonly used temporal signal-to-noise ratio (tSNR) weighted by the square root of the number of samples. We computed this functional sensitivity metric both at the whole-brain level, restricted to grey matter tissue, and within three regions-of-interest (ROIs) located beneath the anodal electrode, the cathodal electrode, and an independent site remote from the electrodes, which was used as a reference. This was done for each of the three stimulation conditions (i.e., No-tDCS setup in place, Sham-tDCS, and Anodal-tDCS). The t-score testing for the mean signal was computed using the Statistical Parametric Mapping software (SPM12) running under Matlab 2020a (MathWorks, Natick, MA). Matlab scripts for the analyses carried out can be accessed here ( Nardo *et al.*, 2021a; https://doi.org/10.5281/zenodo.7569833). Matlab scripts are largely compatible with and may be run in the open source alternative GNU Octave.

### fMRI acquisition

MR data were collected using a 20-channel head coil on a 3T Siemens Prisma ^Fit^ system (Siemens, Erlangen, Germany) at the WCHN. Data included a T1-weighted MPRAGE acquisition for anatomical reference (TR = 2.53 s, TE = 3.34 ms, voxel size = 1 × 1 × 1 mm ^3^, field of view = 256 × 256 × 176 mm ^3^), field maps of the B _0_ field inhomogeneity and corresponding voxel displacement maps were derived from a dual echo gradient-echo acquisition. This field map was subsequently used to apply distortion correction to the functional images, which were T2*-weighted echo-planar images (EPI) and acquired with the following parameters: TR = 3.36 s, TE = 30 ms, 48 axial slices with ascending slice ordering, slice thickness = 2.5 mm, inter-slice gap = 0.5 mm, in-plane resolution = 3 × 3 mm, flip-angle 90°. A total of 70 volumes (65 of interest and 5 dummies) were acquired in each of three consecutive runs, lasting approximately 4 minutes each. There was no task beyond the use, or not, of the tDCS stimulation.

### fMRI preprocessing

Functional data were preprocessed and analysed in native space as defined by (i.e., following co-registration to) the anatomical image using the Statistical Parametric Mapping software (SPM12) running under Matlab 2020a (MathWorks, Natick, MA). All functional volumes of interest (the first five volumes were discarded to allow the magnetisation to reach steady state) were realigned and unwarped using the session-specific voxel displacement maps derived from the B _0_ mapping data. The structural image was segmented into grey matter, white matter, and cerebrospinal fluid.

### fMRI analysis

Statistical analyses were performed in a run-specific fashion. Parameter estimates were calculated for all brain voxels using the General Linear Model (GLM) in SPM12. To remove any low-frequency scanner drifts, data were high-pass filtered using a set of discrete cosine functions with a cut-off period of 128s. A GLM consisting of the nuisance regressors describing motion-related variance (given the experiment was task-free) and temporal autocorrelation was evaluated, and the t-score testing for the mean signal extracted.

### Regions-of-interest (ROIs)

**Figure 1.**
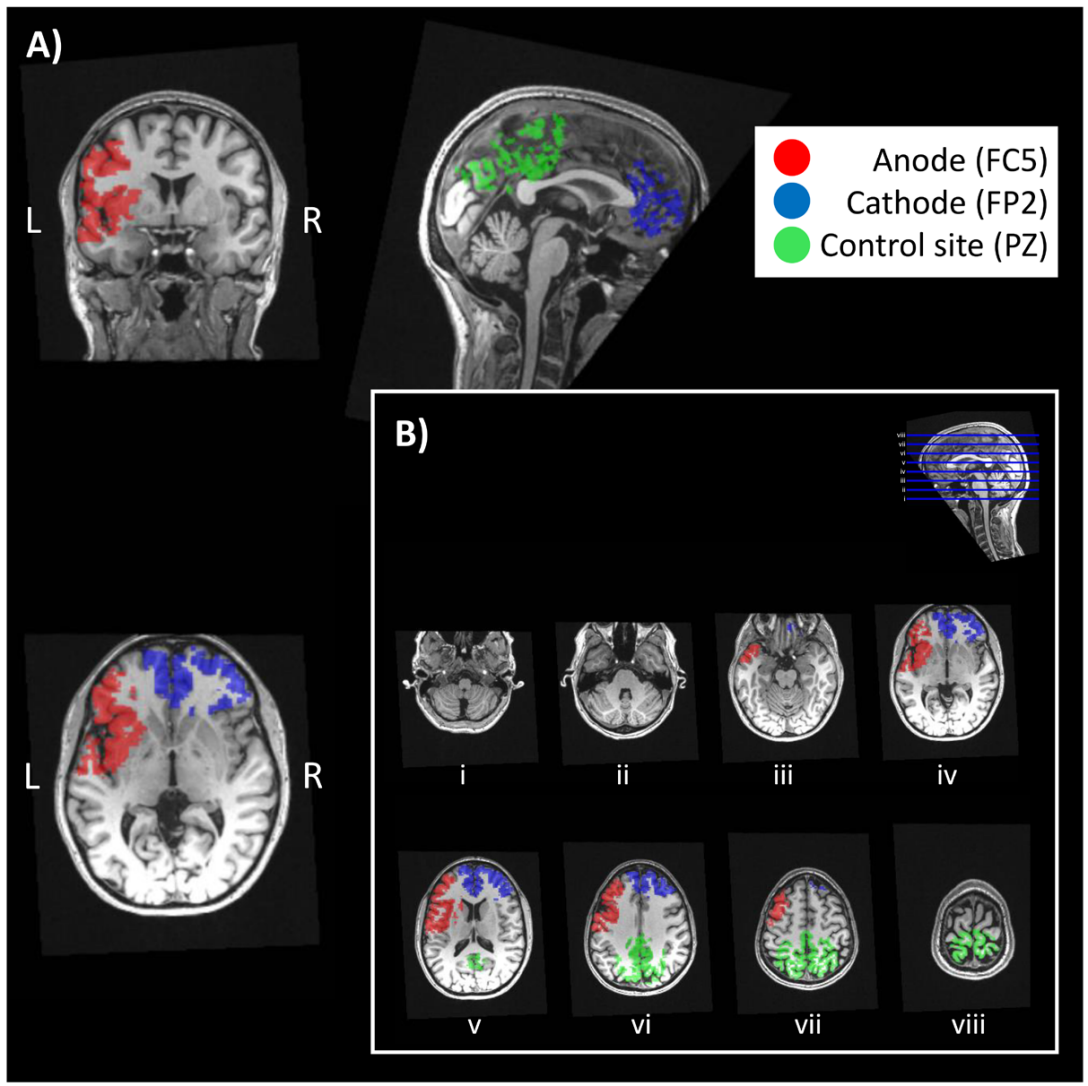
Regions-of-interest (ROIs). **A**) Locations of the three ROIs tested overlaid on the coronal, sagittal, and axial sections of a T1-weighted image. ROIs were defined beneath the anodal electrode (in red, corresponding to FC5 in a 10-20 system), cathodal electrode (in blue, corresponding to FP2) and at an independent site remote from the electrodes, used as a reference (in green, corresponding to PZ). **B**) Locations of the three ROIs tested overlaid on the axial sections shown in Figure 2.

To test more specifically for any local changes within the vicinity of the stimulating electrodes, a series of regions of interest (ROIs) were created in two steps. First, spheres with a 40 mm radius were created around the cortical projections (using the anatomical image as a reference) of the anode electrode (corresponding to FC5 in a 10-20 system), cathode electrode (corresponding to FP2), and an independent site remote from both electrodes as a reference (corresponding to PZ), using the MarsBaR toolbox for SPM. Second, each of these spherical ROIs (in native space) was (inclusively) combined with the whole-brain segmented grey matter tissue from the T1-weighted image. Figure 1 illustrates the locations of the three ROIs tested overlaid on axial brain sections.

## Results

**Figure 2.**
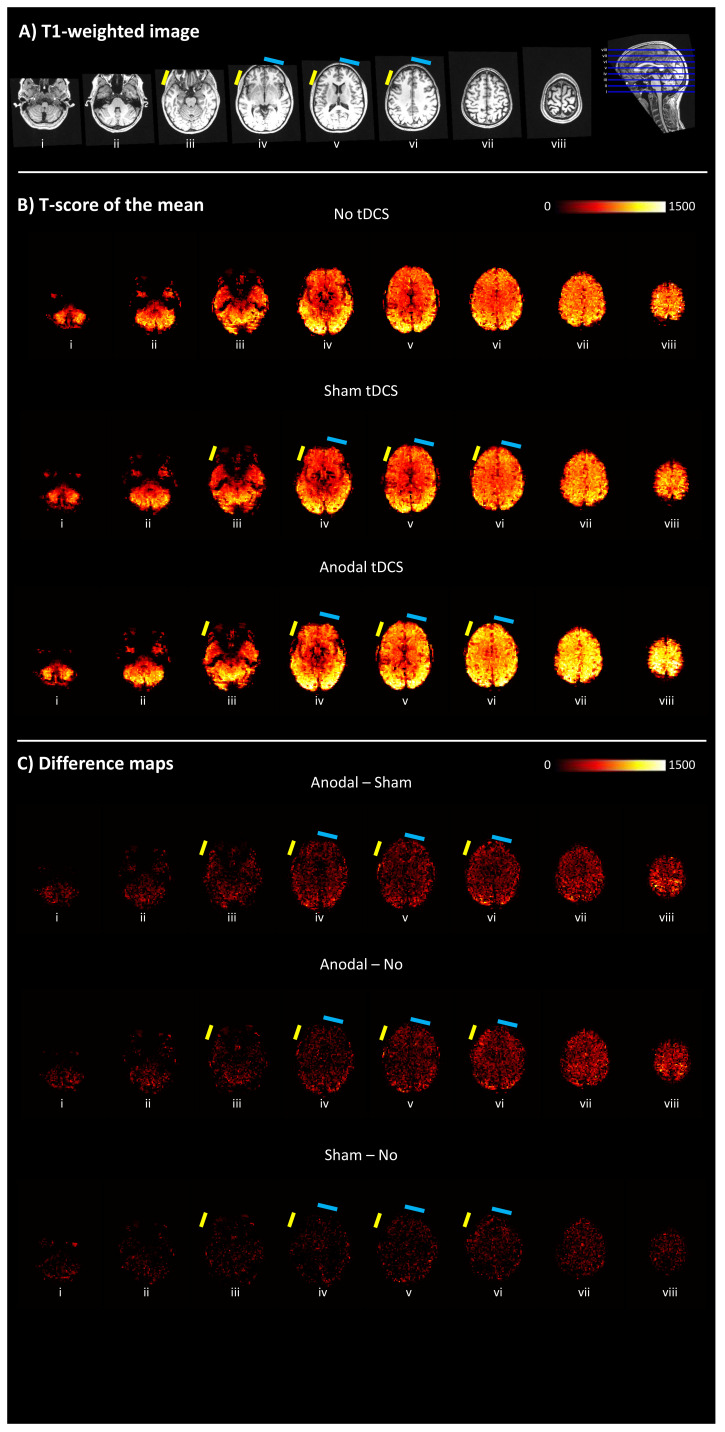
Quality assessment of MR images acquired on one participant. **A**) High-resolution (1 mm, isotropic) structural T1-weighted image denoting electrode locations shown as a reference. The approximate position of the electrodes is indicated by the coloured small rectangles (anodal = yellow; cathodal = light blue). **B**) t-score of the mean maps. In all panels, axial slices (in ascending order from *i* to *viii* with location denoted by the blue lines on the sagittal section in A) are 15 mm apart. There are no visible signal dropouts or distortions introduced by the electrodes and/or conductive paste. The functional sensitivity measures (t-scores of the mean) are comparable across stimulating conditions, though highest in the Anodal-tDCS case. **C**) Difference maps between conditions.

Quality assessment of whole brain MR images indicated no visible signal dropouts or image distortions in the brain introduced by the tDCS electrodes, equipment and/or conductive paste ( Nardo *et al.*, 2021a). This was the case for both the high-resolution (1 mm, isotropic) structural T1-weighted image and, more notably, the EPI images across the three conditions (No-tDCS, Sham, and Anodal stimulation). The functional sensitivity measure (i.e., t-scores of the mean) was broadly comparable at a whole-brain level across the three conditions. This indicates that functional MR sensitivity was not degraded or adversely affected by the tDCS set-up and stimulation protocol. These results are displayed in Figure 2.

**Figure 3.**
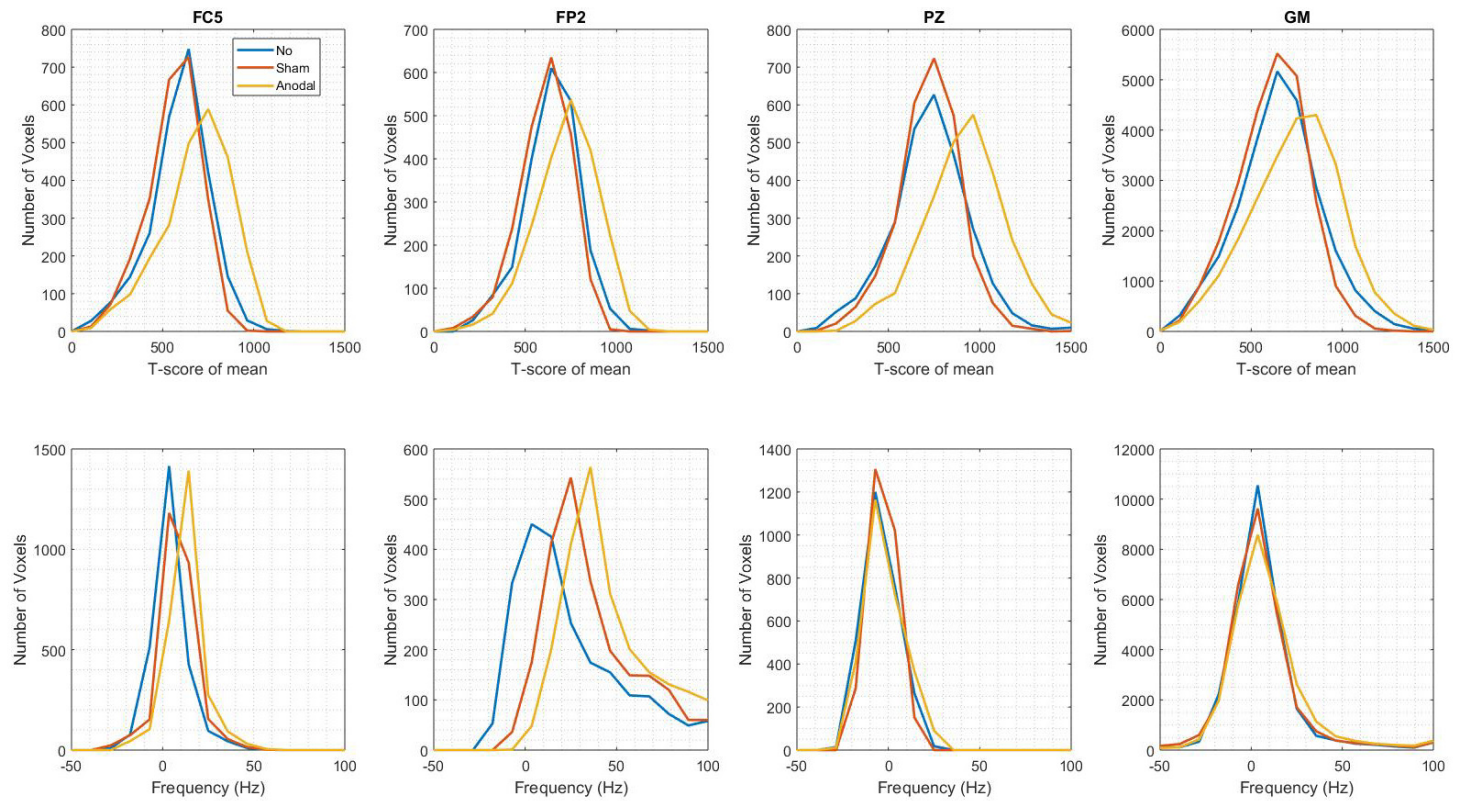
Functional sensitivity metrics across conditions in the three regions of interest (ROIs) (from left to right: anodal electrode – FC5, cathodal electrode – FP2, independent site remote from the electrodes – PZ) and in grey matter (GM), (rightmost column). **Top row:** t-score of the mean extracted from the GLM used for the functional analyses. This provides a tSNR-like measure while additionally accounting for scanner drift, motion-related variance, and temporal autocorrelation. Kolmogorov-Smirnov tests identify no significant differences between the distributions across conditions (cf. Table 1). However, the t-score of the mean is higher in the Anodal-tDCS condition. This discrepancy is attributed to positioning differences, whereby the participant was closer to the coils in this condition due to the absence of a cushion at the crown of the head. The higher proximity to the coil leads to higher signal because of the higher receiver sensitivity. **Bottom row:** off-resonance frequency (in Hz), which measures the degree of field inhomogeneity. A shift to higher frequency is observed for the anodal (FC5) and cathodal (FP2) electrodes, indicating a field offset in these regions. However, Kolmogorov-Smirnov tests identify no significant differences between the distributions across conditions (cf. Table 1).

Region-Of-Interest (ROI) analyses investigated the frequency distribution and t-score of the mean values extracted from the grey matter (GM) anode (FC5), cathode (FP2) and reference (PZ) ROIs. The results illustrated in Figure 3 found there was a high level of overlap for the t-score of the mean distribution values in the Sham- and No-tDCS conditions extracted from each of the ROIs. A shift to higher t-score of the mean values was evident for the Anodal-tDCS case. The width of the frequency distributions in each ROI, reflecting field inhomogeneity, was not increased across conditions indicating that no field inhomogeneity was introduced by the tDCS equipment or stimulation condition (see Table 1). In the cathode ROI (FP2), a shift to higher frequency offsets, as well as greater inhomogeneity (higher IQR), was observed across all stimulation conditions, indicative of poorer field homogeneity and greater difficulty shimming in this ROI.

**Table 1.** **Top:** descriptive statistics (median and interquartile range) to characterise frequency distributions for each metric (t-score of the mean, off-resonance frequency), in each ROI (FC5, FP2, PZ, and grey matter – GM), and for each stimulation condition (No-tDCS, Sham-tDCS, Anodal-tDCS). **Bottom:** results (p-values) of the two-sample Kolmogorov-Smirnov tests to test the null hypothesis that the distributions across conditions came from the same continuous distributions. None of the statistical comparisons returned a significant result.

ROI		FC5		FP2		PZ		GM	
metric	descriptive stats	median	IQR	median	IQR	median	IQR	median	IQR
**t-score**	***No***	609	189	656	180	734	253	661	283
	***Sham***	580	184	620	181	732	215	632	259
	***Anodal***	710	245	743	227	925	282	761	332
**frequency**	***No***	4.4	9.5	15.3	37.6	-4.4	13.0	3.7	15.7
	***Sham***	8.0	8.7	29.7	32.6	-3.1	10.0	2.6	16.5
	***Anodal***	12.3	8.9	39.3	31.5	-3.5	14.7	5.0	19.8
ROI		FC5		FP2		PZ		GM	
**t-score**	***No vs. Sham***	0.678		0.954		0.508		0.241	
	***Sham vs. Anodal***	0.841		0.678		0.241		0.241	
	***No vs. Anodal***	0.996		0.841		0.954		0.954	
**frequency**	***No vs. Sham***	0.996		0.996		0.996		0.954	
	***Sham vs. Anodal***	1.000		0.996		0.841		0.954	
	***No vs. Anodal***	1.000		0.841		1.000		0.841	

In order to formally compare the differences between the stimulation conditions, for each of the assessment metrics (i.e., t-score of the mean and off-resonance frequency), and in each ROI, a two-sample Kolmogorov-Smirnov test was used to test the null hypothesis that the distributions across conditions (e.g., Anodal-tDCS vs. No-tDCS) came from the same continuous distributions. The results of the statistical comparisons are summarised in Table 1.

In summary, taken together these whole-brain and ROI results indicate functional sensitivity was not degraded, nor was field inhomogeneity introduced by, the tDCS equipment or stimulation conditions.

## Discussion

Combining non-invasive neuro-stimulation and functional neuroimaging techniques can provide a unique opportunity to understand the immediate and long-lasting effects of tDCS on the brain. At the WCHN, tDCS is being used alongside fMRI in order to understand the neural mechanisms underlying tDCS’ behavioural effects. The combination of fMRI and tDCS methods, including simultaneous/concurrent tDCS-fMRI can provide unique insight into the neuromodulatory effects of tDCS not only in the targeted brain regions, but also their interconnected networks. The ultimate aim of these mechanistic experiments is to find a relationship between behavioural and neural responses to tDCS. Here, we present our detailed procedural methodologies with the aim of increasing replicability of tDCS-fMRI methods and the reliability of results for future studies. We hope this will in turn enable the field to gain greater insight into the mechanisms underpinning neural and behavioural modulation by tDCS, which would open up new directions within scientific research and clinical applications, such as developing targeted and meaningful therapies.

In this section, we first focus on discussing the identified safety risks and accompanying risk mitigations that are specific to the incorporation of the tDCS equipment into the MRI environment that were considered critical during the writing of this operational procedure. Then we discuss the specific challenges of concurrent tDCS-fMRI and how acquisition of appropriate B _0_ field mapping data can help allay concerns over artefacts and false positive functional results from perturbation of the magnetic field. To date, this protocol has proved a safe and reliable means of obtaining high quality fMRI data concurrently with the application of 2 mA anodal tDCS (20 minutes) in over 18 healthy older adults and 36 aphasic stroke patients ( Nardo *et al.*, 2021b; Ondobaka *et al.*, 2020).

It needs to be stressed that the procedures, safety measures and image quality issues discussed in the present work are specific to conventional tDCS configurations and frontal montages, which may need adjustment for substantially different set-ups (e.g., high-definition-tDCS configurations, anterior-posterior tDCS montages, etc.). Given the above-mentioned impact of research questions, methods and parameters variability on tDCS studies (cf. Introduction), we cannot assume that the present procedures and validation results can be generalised to other types of tDCS protocols with different characteristics, for which targeted further studies are needed. We should also point out that the safety and image quality considerations discussed below overlap with some of the key factors identified by Ekhtiari *et al.* in their checklist for assessing the methodological quality of tES-fMRI studies ( Ekhtiari *et al.*, 2022). Indeed, this recently published consensus guide represents a notable step toward increased standardisation, inter-Lab communication, methodological transparency and reproducibility of tES-fMRI studies across a variety of contexts. Building on this, the present work translates Ekhtiari *et al.*’s guidelines into practice for a specific tDCS-fMRI setup.

### Safety considerations for tDCS in the MRI environment

The MRI environment poses a number of significant risk factors, primarily due to the various comparatively strong magnetic fields used, which can vary in both space and time. The main magnetic field (3 Tesla in our case) can exert significant forces on ferrous objects or current-carrying conductors. The primary risk to be mitigated against is the introduction of any ferrous components. The NeuroConn DC stimulator Outer-Box contains an RF filter with ferrous components. This Outer-Box should never enter the Scan Room to prevent it from becoming a projectile under the force exerted by the main magnetic field. It should be housed within the waveguide and always be placed and removed via the Control Room.

The time-varying magnetic fields used to achieve the excitation process in MRI have associated electric fields (i.e., this is an electromagnetic RF field). These can induce current flow, both in the participant and any equipment, and lead to heating. During scanning, the MRI system continually monitors the transmitted power to ensure it is as expected, and within regulatory limits by modelling the specific absorption rate (SAR), i.e., the energy deposition in the participant, in the absence of any equipment. To accommodate the introduction of the tDCS equipment, and ensure it does not invalidate the model, further mitigation strategies are adopted. Care is taken to arrange cables without introducing any closed loops in which current could flow, and the electrode leads are run along the centre of the bore using the bespoke foam-base. This base has a groove that maximises the safety of the cabling configuration by ensuring it is parallel with the bore, centred within the transmitting RF coil’s volume without any loops, and running away from the participant. The tDCS equipment also has multiple RF filters incorporated, and a high input impedance to minimise the currents that could flow as a result of any induced voltage, which could also be caused by the rapidly switching imaging gradients. Resistors are incorporated into the leads adjacent to the electrode pads to further limit any possible current flow. The MRI compatible electrodes are made from an electrically conductive rubber. It is possible that circumferential RF currents could be set up directly within these relatively large pads but, at least for the low SAR sequences used here, heating is negligible, as confirmed by previous experiments ( Holland *et al*., 2011). As a further risk mitigation strategy, in our Lab only low power imaging sequences are used.

The MRI scanner is situated within a Faraday cage, a continuous copper foil on a wooden support structure, designed to be impermeable to RF fields and often referred to as the RF cage. The purpose of this cage is to contain any internally-generated RF sources, e.g. the transmit coil, within the Scan Room and to prevent any external sources from the everyday environment, which could reduce image quality, from entering the Scan Room. The RF cage is explicitly grounded at a single point to prevent unintended connections to the building’s electrical ground. To preserve this condition, all electrical connections to the cage are made via a so called “penetration panel” and are RF filtered to maintain the intended RF isolation. Most RF filters used for this purpose are formed of capacitors and inductors in which the capacitors are connected to the RF screen of the cage, which is itself connected to ground. This means that the filters provide a pathway to ground. If any equipment entering the Scan Room in this way is connected to the participant, they too become part of a grounded circuit. The tDCS equipment has been designed for stimulation of human participants in the MRI environment according to the International Electrotechnical Commission standards (60601-1 Class 1 [battery powered] with Type BF [Body Floating; i.e., no possible route to ground] applied part standard). The NeuroConn manufacturer achieves this by using electrically insulated non-grounded filters, which ensure that the participant being scanned is not connected to ground. This is an intrinsically safe arrangement because, even if a fault condition were to develop during scanning, such that the participant was brought into contact with high voltages, no conducting path is available for a dangerous current to flow through the participant to ground via the tDCS apparatus. To maintain this BF safety status, the penetration panel has not been used to integrate the tDCS equipment in our Lab.

The other means of penetrating the RF cage is via a waveguide – a long cylindrical tube that will only allow signals above a certain frequency, known as the cut-off frequency, to pass and therefore can be used to exclude RF signals that would otherwise interfere with imaging. This is the approach we have used to integrate the tDCS equipment into the scanner environment. While this approach ensures that the equipment and participant are not connected to ground, it also introduces the risk of violating the RF isolation (a data quality requirement) and allowing RF from the external environment into the Scan Room.

Two filters are used to attenuate current flow: the MR-compatible “Inner-Box” minimises any currents flowing in the section of the electrode lead in the bore, while the MR-incompatible “Outer-Box” in the waveguide itself prevents current flow in the outer cabling entering the Scan Room. However, these RF filters have limited performance meaning that a risk of RF interference compromising the imaging remains, particularly if there is an equipment fault. This data quality risk motivated the extension of our RF cage to enclose the entire stimulator within a shielded box attached, via shielded flexible metallic tubing, to the outside of the waveguide. This additional box has a removable shielding lid, which creates a robust RF seal via fingerstrip gaskets (e.g., https://hollandshielding.com/Shielding-gasket-solutions-materials#Fingerstrips) and a viewing aperture that is small enough not to compromise the RF shield but sufficient for operation and monitoring of the tDCS device. The lid also proves a useful means of ensuring that the researcher can remain blind to the experimental tDCS condition.

The electrical signal used by the trigger input to drive the stimulator is galvanically isolated from the rest of the circuitry by the manufacturer (cf. NeuroConn manual). When the box is manually triggered, this isolation can be verified by visual inspection since no wires are connected to anything that could be grounded. In theory, an electrical cable can normally be connected directly from the controlling computer, as long as the galvanic isolation is certain. However, in our case such a connection would also have compromised the additional RF screening by providing a path for RF current to flow. Therefore, a fibre-optic trigger signal from the Stimulus PC enters the shielded box through a small waveguide and is subsequently converted to an electrical signal, via battery-control, to drive the stimulator.

### tDCS and fMRI image quality and safety control study

Prior to any neuroscience experiments, and in particular due to the extension to the RF cage, the tDCS equipment setup should be tested to ensure that the integrity of the RF cage isolation had not been compromised. In our case, this was done by measuring the cross-talk between two adjacent MRI scanners with and without the tDCS equipment in place. These tests confirmed that, with the RF shielding solution employed here, the RF noise level was equivalent regardless of the presence of the tDCS equipment. As with any experimental setup, routine quality assurance should also be employed. All equipment should be regularly inspected for damage and maintained in keeping with manufacturer guidelines.

The introduction of an electrical current into the scanner’s magnetic field results in further warping of the magnetic field (i.e., field artefact). This artefact is of critical concern for BOLD fMRI protocols, as it may result in false positive patterns in BOLD signal ( Antal *et al*., 2014; Woods *et al*., 2016). Online tES-fMRI studies are therefore more susceptible to artefactual noise than other fMRI scenarios, the magnitude and nature of which are likely to depend on the exact experimental setup within each Lab, for each experiment, across participants (cf. Weiskopf *et al.*, 2009, for related considerations using TMS). We believe that careful experimental designing is an essential factor here. This highlights the importance of having a replicable set-up with properly placed and shielded electrode cables and stimulation equipment within the scanning area. For example, one study demonstrated evidence of BOLD signal within brains of two cadavers during a concurrent tDCS and fMRI protocol ( Antal *et al*., 2014). Whilst a previous study from our Lab demonstrated visual evidence of change in echo-planar imaging (EPI) field maps that was limited to the scalp/surface near to the electrode site ( Holland *et al*., 2011). These contrasting cases demonstrate the need for careful consideration of concurrent tDCS-fMRI data, and acquisition of appropriate field map data to allay concerns over false positive functional results from perturbation of the magnetic field.

**Figure 4.**
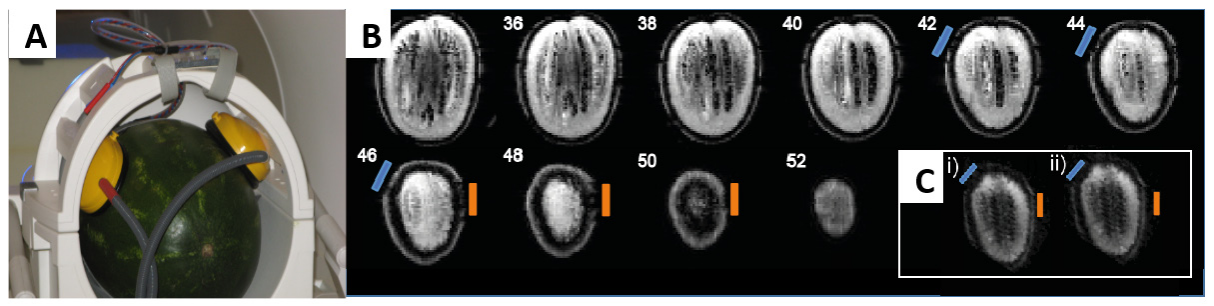
Control concurrent tDCS and fMRI study. ( **A**) A watermelon of similar size to a human adult head was chosen as a continuous 2 mA anodal DC could be passed through the surface. The headphones and tDCS electrodes were positioned on the object in the same orientation, and with the same tDCS/fMRI set-up as was used for the main human study (cf. Holland *et al*., 2011); ( **B**) Multi-slice coronal view of the watermelon field distortion. The blue bar indicates the location of the anode electrode, orange the cathode electrode where mild perturbation of the signal is evident in slices 42-50 at the surface layer of the watermelon under each electrode; ( **C**) EPI from slice 46 (watermelon data) for (i) un-stimulated (sham) and (ii) stimulated (A-tDCS) fMRI runs. This figure has been used with permission from Holland *et al*., 2011.

To address this, prior to scanning human participants, we recommend a control tDCS concurrent with fMRI study for evaluation of the set-up in each Lab. The purpose of the control study is twofold: (i) to ensure the safety of concurrent tDCS and fMRI, and (ii) to quantify any noise effects in the images induced by tDCS delivered simultaneously with the task stimuli. For example, in a previous experiment we delivered 2 mA anodal tDCS stimulation for 20 minutes concurrently with the identical stimulus delivery set-up as used in the main study to an inert object (a watermelon; cf. Holland *et al*., 2011). Results indicated that: (i) during stimulation no significant changes in surface temperature were detected over time; and (ii) in distortion correction field maps only minimal perturbation of signal was observed at the electrode site (see Figure 4).

The signal distortion was restricted to the surface of the watermelon only. Processing of the acquired functional data found no effects of tDCS on sham or stimulated runs. Together, these results indicated no imaging artefacts induced by the tDCS/fMRI set-up that could account for the effects of Anodal-tDCS reported in the subsequent human experimental study (cf. Holland *et al*., 2011 for full details of effects of tDCS on EPI data). A control study such as this, comparing fMRI data for a short duration of time under two tDCS conditions, e.g. anodal vs. sham, can be adapted for any experimental paradigm and tDCS-fMRI set-up.

In the human validation study presented here, the variability introduced by the tDCS equipment appears to be far below the level of variability arising from participant repositioning, reaffirming the great importance of careful positioning. Generally higher t-score of the mean values were observed for the Anodal-tDCS case (cf. Figure 1 and Figure 3). However, given that we would not expect an increase in functional sensitivity when using tDCS, this is more likely to originate from variability in participant repositioning, and scanner adjustments, across the different experimental conditions. Indeed, in the Anodal-tDCS case, a cushion was inadvertently not placed at the crown of the head, resulting in the participant being positioned furthest into (superiorly) the sensitive volume of the receiving coil, boosting sensitivity. Receiver coils have a non-uniform sensitivity profile that decreases with distance from the coil. We therefore attribute the higher sensitivity observed in the Anodal-tDCS case to the greater proximity of the participant to the coil that was specific to this case. The cathode ROI (FP2) had greatest field inhomogeneity regardless of imaging condition. A frequency offset was observed in this ROI for the Anodal-tDCS case, however, there was no broadening of the frequency distribution. This indicates that the introduction of the tDCS equipment did not increase the field inhomogeneity. In fact, the broadest distribution was observed in this ROI for the No-tDCS condition. Reiterating the critical issue of participant repositioning inside the scanner, a better option for the No-tDCS (i.e., control) condition would perhaps have been to only have the electrodes attached but with no cables/stimulator connected enabling a more consistent comparison between different conditions.

## Conclusion

In this paper, we deliver the Wellcome Centre for Human Neuroimaging standard operating protocol ( SOP) for technically sound and safe application of tDCS concurrently with fMRI. Although the MR-compatible tDCS technique is seemingly simple and easy to apply, we discussed specific aspects that must be taken into consideration when integrating the approach into an MRI environment to obtain both a safe experimental set-up and reliable results with maximal image quality. This SOP and the experimental data validating its efficacy is provided as a detailed framework to systematically report the main technical elements of tDCS-fMRI, which can be adopted and used as a baseline for prospective real-world applicability. It is hoped that this will enhance the quality of tDCS-fMRI application in future studies, help provide practical solutions to the technical challenges and complications of the method, and therefore improve the quality of scientific work in this field further.

## Data Availability

Zenodo: WCHN/tDCS_fMRI: tDCS for fMRI SOP Release. https://doi.org/10.5281/zenodo.7569833 ( Nardo *et al.*, 2021a). This project contains the following underlying data: Andodal_tDCS MPRAGE No_tDCS Sham_tDCS Zenodo: WCHN/tDCS_fMRI: tDCS for fMRI SOP Release. https://doi.org/10.5281/zenodo.7569833 ( Nardo *et al.*, 2021a). This project contains the following underlying data: WCHN_tDCS_fMRI_SOP.pdf (Standard operating procedures)

## Software availability

- Source code available from: https://github.com/WCHN/tDCS_fMRI/tree/v1.1.0
- Archived source code at time of publication: https://doi.org/10.5281/zenodo.7569833  ( Nardo *et al.*, 2021a).
- License: GNU General Public License v3.0
