# Supplementary material for: Transcranial direct current stimulation with functional magnetic resonance imaging: a detailed validation and operational guide
Source: Wellcome Open Res. 2023 Feb 6;6:143. Originally published 2021 Jun 7. [Version 2] doi: 10.12688/wellcomeopenres.16679.2 (PMC10050906; doi:10.12688/wellcomeopenres.16679.2)
Supplement: Peer review history

# Peer review: Reviewer response for version 2

**DOI:** 10.21956/wellcomeopenres.21018.r60585

**Published:** 2023-07-17

**Authors:** Lars G Hanson

**Affiliations:** 1 DTU Health Tech, Technical University of Denmark, Kgs. Lyngby, Denmark; 2 Copenhagen University Hospital Amager and Hvidovre, Hvidovre, Denmark

This study presents safety considerations and quality assurance for a specific tDCS-fMRI setup, exemplified by data from one subject. The considerations are aimed to be generalizable, however. The study is conducted with the help of a shared standard operation procedure (SOP) for tDCS-fMRI, and the example data and analysis software are also shared. The specific tDCS set-up was found to not introduce significant quality differences between conditions (No-tDCS, Sham-tDCS, Anodal-tDCS). This was based on evaluation of t-score of the signal mean and of field inhomogeneities in regions of interest. The SOP and QA evaluation may be valuable for researchers establishing tDCS-fMRI and similar protocols. Some safety aspects need further elaboration, however:

 The manuscript states that "resistors are incorporated into the leads adjacent to the electrode pads to further limit any possible current flow". Such safety resistors (here 5.4 kiloohms) indeed limit the current, but importantly only effectively at the site of the resistor. At the relevant MHz frequencies, standing electro-magnetic waves may occur on either side of a localized resistor inserted in a high-conductivity cable (the resistor will be at a zero-current node). In the present case, the safety resistor is near the head (centimeters), thus preventing standing waves to occur between the head and the safety resistor. However, standing waves may still occur on the other side of the resistor, and the resulting high voltages occurring at the resistor can damage both the resistors (consistent finding for the used equipment), and any nearby tissue. Hence the importance of keeping safety resistors away from tissue should be stressed in the otherwise detailed safety description.

 An alternative approach to avoid standing cable waves altogether is to use distributed impedance instead of localized safety resistors (Gregersen *et al.*, 2021 ^1^). This approach was recently adopted by the manufacturer of the stimulation equipment, and it furthermore allows the overall cable impedance to be reduced such that stimulation significantly above 2 mA current strength can be used during fMRI. Also, distributing the impedance allows the cables to be oriented along the B0 field, reducing the effects of tDCS currents on the MRI quality as discussed in the manuscript (the SOP and the manufacturers safety manual prescribe orthogonal cable orientation near the head to avoid damage to the safety resistors, but currents in this direction distorts the z-component of the magnetic field that MRI relies on).

 Other comments:

 The manuscript states that "the stimulator works with a pre-defined impedance limit ≤10 ohm, above which the device does not operate". Probably it should be 10 kiloohms, or it should be clarified how this fits with the safety resistors alone being 5.4 kiloohms.

 The authors attribute the higher sensitivity observed in the Anodal-tDCS case to the proximity of the participant to the coil that was specific to the Anodal-tDCS case. This may indeed be, but in general, the conductive electrode pads also affect RF fields which may locally increase the coil sensitivities. This effect will be independent of stimulation. Field distortions (B0 and B1) speak in favor of also using electrodes and cables during tDCS-MRI control conditions.

Is the rationale for developing the new method (or application) clearly explained?

Yes

Is the description of the method technically sound?

Yes

Are the conclusions about the method and its performance adequately supported by the findings presented in the article?

Yes

If any results are presented, are all the source data underlying the results available to ensure full reproducibility?

Yes

Are sufficient details provided to allow replication of the method development and its use by others?

Yes

Reviewer Expertise:

MRI methods and applications

I confirm that I have read this submission and believe that I have an appropriate level of expertise to confirm that it is of an acceptable scientific standard, however I have significant reservations, as outlined above.

## References

[1] : Safety evaluation of a new setup for transcranial electric stimulation during magnetic resonance imaging. *Brain Stimul* .2021;14(3) : 10.1016/j.brs.2021.02.019 488-497 10.1016/j.brs.2021.02.019 33706007

# Peer review: Reviewer response for version 2

**DOI:** 10.21956/wellcomeopenres.21018.r54721

**Published:** 2023-03-28

**Authors:** Marius Moisa

**Affiliations:** 1 Department of Economics, Urich Center for Neuroeconomics (ZNE), University of Zurich, Zürich, Switzerland

The authors have addressed all my comments. I would like to congratulate the authors for sharing this SOP for the online combination of tDCS with fMRI. I think this is very useful. I advise all the researchers to take this SOP into consideration when setting up this combination of methods.

Is the rationale for developing the new method (or application) clearly explained?

Yes

Is the description of the method technically sound?

Yes

Are the conclusions about the method and its performance adequately supported by the findings presented in the article?

Yes

If any results are presented, are all the source data underlying the results available to ensure full reproducibility?

Yes

Are sufficient details provided to allow replication of the method development and its use by others?

Partly

Reviewer Expertise:

Online combination of tCS with fMRI, neuroscience, brain stimulation, fMRI.

I confirm that I have read this submission and believe that I have an appropriate level of expertise to confirm that it is of an acceptable scientific standard.

# Peer review: Reviewer response for version 1

**DOI:** 10.21956/wellcomeopenres.18392.r47627

**Published:** 2022-01-24

**Authors:** Marius Moisa

**Affiliations:** 1 Department of Economics, Urich Center for Neuroeconomics (ZNE), University of Zurich, Zürich, Switzerland

In the current study, the authors provide a step-by-step guide through the standard operating procedure that is in place at Wellcome Centre for Human Neuroimaging for the online combination of tDCS with fMRI. These detailed methodological procedures should allow researchers to implement such a setup in a safe, artefact-free, and in a replicable manner. I find this SOP/paper very useful for the interested research community, however, more details and a few clarifications should be addressed before publication.

  

1. The electric field distribution of a standard tDCS montage (like the one implied in the current paper) has a maximum electric field between the two tDCS electrodes. I would recommend the authors, if possible, to simulate the electric field for the applied tDCS montage and perform similar analyses in two additional ROIs:
  In an ROI where the electric field is maximum, i.e. between the two tDCS electrodes.
  In a whole gray matter mask. This is particularly important since the aim of combining brain stimulation with fMRI is to investigate stimulation effects at the whole-brain level.
2. For the condition “No-tDCS setup in place“, means that no tDCS electrodes are attached to the head? In this case, the participant's head was removed from the MR head coil and the position of the head inside the MR coil might be slightly different for the control condition as compared to “sham tDCS” and “anodal tDCS” conditions. Thus the t-maps could be affected by this, e.g. due to different spatial sensitivity of the MR coil. For future tests the authors might consider a better control condition, e.g. only the electrodes attached and no cables/stimulator connected that could make the comparison between different conditions more reliable.  
3. The authors should describe in more detail the setup and the stimulation protocol: size of the tDCS electrodes, sham-tDCS (was it only ramping up and ramping down or also a few seconds of stimulation in between), length of the ramp-up/ramp-down for the anodal-tDCS condition. Is the size of the radius of the ROIs related to the size of the electrodes?
4. I agree with another reviewer that a more systematic way of quantifying the differences between different stimulation conditions should be provided. Thus, these analyses could be applied/reproduced by any other researcher that would like to combine tDCS with fMRI.  
5. Reproducibility issue: If it is not much of an effort, the authors could test if the presented results are validated by the data acquired in a second participant. Or at least discuss this issue.
6. The authors briefly mention the problem of possible false positive functional results due to the tDCS stimulation. However, none of the data/analysis presented in this paper addresses this issue. The authors should discuss this in more detail or address this issue with new sets of data. Based on several sets of data acquired in a watermelon in our lab (we used a similar block design as in Weiskopf et al. 2009 ^1^) this problem always occurs and should be taken into consideration when designing a tDCS-fMRI experiment.

Minor comments:

1. Page 4, Results section: The authors state “no visible signal dropouts or signal distortion”. Is this valid for the brain, if yes the authors should mention this. Based on my experience at least for the setup that we have in our lab (also a NeuroConn setup) we do see static distortion in the skull under the MR-compatible tDCS-electrodes.
2. The authors should discuss the overlap between the current paper and a consortium paper by Hamed Ekhtiari and colleagues ^2^.

Is the rationale for developing the new method (or application) clearly explained?

Yes

Is the description of the method technically sound?

Yes

Are the conclusions about the method and its performance adequately supported by the findings presented in the article?

Yes

If any results are presented, are all the source data underlying the results available to ensure full reproducibility?

Yes

Are sufficient details provided to allow replication of the method development and its use by others?

Partly

Reviewer Expertise:

Online combination of tCS with fMRI, neuroscience, brain stimulation, fMRI.

I confirm that I have read this submission and believe that I have an appropriate level of expertise to confirm that it is of an acceptable scientific standard, however I have significant reservations, as outlined above.

## References

[1] : Image artifacts in concurrent transcranial magnetic stimulation (TMS) and fMRI caused by leakage currents: modeling and compensation. *J Magn Reson Imaging* .2009;29(5) : 10.1002/jmri.21749 1211-7 10.1002/jmri.21749 19388099PMC3077517

[2] : A Checklist for Assessing the Methodological Quality of Concurrent tES-fMRI Studies (ContES Checklist): A Consensus Study and Statement. *medRxiv* .2020; 10.1101/2020.12.23.20248579 10.1101/2020.12.23.20248579 PMC761268735121855

# Peer review: Reviewer response for version 1

**DOI:** 10.21956/wellcomeopenres.18392.r47626

**Published:** 2022-01-13

**Authors:** Ines Violante, Danielle Kurtin

**Affiliations:** 1 School Of Psychology, University Of Surrey, Guildford, UK

In this article, the authors provide a technical guide on how they have implemented a simultaneous tDCS and fMRI protocol. Alongside the article, they provide a detailed SOP and data which can be a helpful resource to researchers combining these techniques, particularly to those who are novel to the field. Together, these resources are important to promote open research practices and replicability of future studies.

 The article focuses on fMRI data from one participant tested across three conditions, no-tDCS, sham-tDCS and anodal-tDCS. Data quality across conditions was investigated in grey matter and in three predefined regions-of-interest (ROIs). The discussion on ‘Safety considerations for tDCS in the MRI environment’ is well achieved and insightful.

 However, we believe that there are a number of points that could be addressed to enable others to make full use of the methodology presented in this article. We have highlighted our main questions below (minor issues are summarised at the end).

1. While the article is presented as focusing “on the methods required for the safe use of tDCS equipment in the MRI environment” both the article and SOP only focus on the usage of one of the multiple tDCS equipments that are available for combined tES+fMRI experiments. Specific methodologies will vary depending on the equipment used. The Introduction should make this clearer.
2. Though the authors point out there is a gap in the literature regarding studies that quantify the influence of tDCS-fMRI on the magnetic field, they do not introduce the two metrics used in this study that assess image quality across stimulation conditions. The Introduction would be strengthened by providing a rationale for using the author’s chosen metrics.
3. In the “tDCS equipment and procedure” section the authors state they “ensure the participant was happy with the stimulation sensation.”. Could the authors provide more information on how this was assessed? Perhaps information on what is acceptable as tolerable could be added to the SOP?
4. More information on the ROIs would be useful. In particular: 1) what is the rationale for the ROI size? 2) how were the coordinates for Pz defined (our understanding is that the montage only included two electrodes that were used for the FP2 and FC5 ROIs)?; 3) what is the rationale to choose Pz as the control ROI?
5. Can the authors provide more details about the electrode size, shape and how the location was defined on the participant in the “tDCS equipment and procedure” section? Here can the authors also provide information about the sham acquisition (i.e. duration of ramp-up/down, current intensity, etc.) and the impedances used across sham and anodal acquisitions?
6. Can the authors provide more information about how data analyses were performed for the sham condition (i.e. did it include ramps)?
7. An important issue with the current version of the manuscript is that there are no statistical analyses to support the statements of lack of differences across conditions. How could a researcher trying to follow your protocol define whether the data quality is acceptable? If the t-mean scores for anodal were left skewed instead of right should researchers assume that their data is contaminated with noise? Recommendations on what would be acceptable deviations from t-means and central frequencies would be more useful to support replications.
8. The discussion introduces the issue of variability associated to participant repositioning. If variation in the participants’ positioning within the coil resulted in their offset T-score of the mean signal, what measures do the authors suggest to prevent such variability in future studies? For example, Person 3 in the detailed SOP is responsible for participant positioning- do the authors think there should be added guidance for Person 3 on where participants should be placed, and how they check positioning?
9. In a similar vein to what the authors intended here, other efforts have been done to summarise previous simultaneous tES+fMRI studies and provide researchers with a checklist for assessing the methodological quality of such studies (see Ekhtiari *et al.*, 2020 - medRxiv 2020.12.23.20248579). ^1^ It could be useful to reflect on whether the safety and image quality considerations the authors highlight are addressed by this checklist.

**Minor:**

1. In the first paragraph of the Introduction it is stated that the techniques uses currents 1-2 mA and durations of up to 20 min. There is however a great variability of protocols that can be employed depending on research questions. Please rephrase these initial sentences.
2. To avoid confusion, the authors should standardize how they refer to the already published SOP (Callaghan, 2021) and this manuscript, as the current manuscript is described both as an SOP and as a guide to the previously published SOP.  
3. Add references (presumably Holland *et al.,* 2011 and Ondobaka *et al.,* 2020) for the studies cited in the final line of the first section of the Discussion.
4. Discussion, Safety considerations for tDCS in the MRI environment: After “specific absorption rate” add “(SAR)”. 

Is the rationale for developing the new method (or application) clearly explained?

Yes

Is the description of the method technically sound?

Yes

Are the conclusions about the method and its performance adequately supported by the findings presented in the article?

Yes

If any results are presented, are all the source data underlying the results available to ensure full reproducibility?

Yes

Are sufficient details provided to allow replication of the method development and its use by others?

Partly

Reviewer Expertise:

neuroscience, brain stimulation, tES, neuromodulation, neuroimaging

We confirm that we have read this submission and believe that we have an appropriate level of expertise to confirm that it is of an acceptable scientific standard, however we have significant reservations, as outlined above.

## References

[1] : A Checklist for Assessing the Methodological Quality of Concurrent tES-fMRI Studies (ContES Checklist): A Consensus Study and Statement. *medRxiv* .2020; 10.1101/2020.12.23.20248579 10.1101/2020.12.23.20248579 PMC761268735121855

# Peer review: Reviewer response for version 1

**DOI:** 10.21956/wellcomeopenres.18392.r44302

**Published:** 2021-06-29

**Authors:** Priyanka Shah-Basak

**Affiliations:** 1 Medical College of Wisconsin, Milwaukee, WI, USA

The authors provide a detailed guide on how to implement tDCS concurrently with fMRI, evaluate the effects of tDCS on MR images and artefacts in 1 healthy volunteer, and discuss safety risks and risk mitigation strategies. The goal for such detailed guide and validation is to minimize inconsistencies across tDCS for fMRI protocols adopted at different institutions around the world, and in turn improve the quality of research involving tDCS-fMRI applications.

 The report is very well-written and informative, especially for labs just starting out and interested in incorporating tDCS+fMRI for the first time. I also value the authors’ rigor in the development of the tDCS for fMRI SOP as well as for providing access to the data that they collected as part of this study to the wider scientific community who may be interested in delving deeper.

 My comments below are minor and focused primarily on improving the readability of the Introduction, and the depth of reporting in the Results. I also have a few clarification questions that the authors should address. I am not an imaging expert and therefore my comments overall are limited to the evaluation of tDCS more generally. I would recommend that, if not already done, an imaging expert should review the relevancy of different measures that are used in this study to evaluate image quality with and without tDCS, and confirm the authors’ interpretation of the results.

**Introduction**

 The authors mention the use of tDCS for up to 20 minutes. However, there are several recent articles that have increased the stimulation duration to up to 40 minutes outside the scanner [e.g., review on chronic pain by Pinto *et al. *(2019) Transcranial direct current stimulation as a therapeutic tool in chronic pain. J ECT]. ^1^ The authors should update this to a range from 20-40 minutes, to be consistent with recent studies using tDCS.

 The authors mention that several guides already exist on the concurrent implementation of tDCS and MRI. But there is still a lack of methodological consensus of tDCS-fMRI procedures between studies. While I agree with the authors that methodological variability exists and can hamper large-scale meta-analyses, the constraint, in my opinion, is not the availability of operational guides (because as the authors pointed out there are a couple of them out there already), but in fact the variability in research questions that different labs around the world are attempting to address using tDCS-fMRI. The parametric space of tDCS is large, involving decisions regarding (but not limited to) the location (target), dose, polarity, training during tDCS, and the use and type of control conditions. These are typically dependent on the research questions of interest, the population of interest, and the current theories related to the neurophysiological mechanisms of interest. Thus, the implication that a manual or an operational guide could help reduce tDCS-related methodological variability and improve replicability of findings is weak, considering that there might still be large methodological variability, stemming from the decisions regarding tDCS parameters and their influence on the BOLD response. For example, we know that the effects of tDCS are highly influenced by the brain state during stimulation and thus regardless of methodological overlap the extent of task load during stimulation could change BOLD activity and introduce another ‘layer’ of variability that cannot be addressed by following the step-by-step procedures laid out in this paper.

 That said, I do think there is a need for better operational guides with procedural steps that are as detailed as the one provided in this paper, to minimize or rule out differences in methodology in tDCS-fMRI implementation as being the main source of significant findings. I believe the authors need to make this point much clearer in the Introduction, given the perplexity related to the variability between tDCS protocols.

 The other point that is not clear from the Introduction is the type of tDCS configuration (conventional or high-definition [HD]), and montages that the authors used in their validation studies. On a related note to the earlier point, the authors should comment on the generalizability or external validity of their procedures and validation results to other types of tDCS protocols, involving different regional targets, polarity, and configurations. I assumed before reading the Methods that the procedures described are specific to conventional sponge-rubber electrode configurations with a reference or cathodal electrode placed on a cephalic site. But this should be described earlier in the Introduction.

**tDCS equipment and procedure**

 How was the determination that the participant was *happy* with stimulation sensation made? Was this a yes/no question that the authors asked?

 I suggest replacing “happy” with “tolerate”.

**Results**

 The authors mention that the “functional sensitivity scores were broadly comparable at a whole-brain level across the three conditions”. How is comparable quantified here? Did the authors run a statistical test to evaluate differences between no-tDCS and sham-tDCS, and no-tDCS and anodal-tDCS, and anodal-tDCS and sham-tDCS? It would help if Figure 2 also included a statistical map comparing the no- and sham-tDCS with the anodal-tDCS condition (or a difference map between stimulation conditions).

 ROI analyses: The authors should run a statistical test to quantify the degree of overlap of t-score distributions across different stimulation conditions. Otherwise, I am not clear how the authors have described the ‘high’ level of overlap, given a shift in values for the anodal-tDCS condition, not found for the other two control conditions. Visually, there does not appear to be a high degree of overlap in top panels in Figure 3.

 Please provide the width of the frequency distribution in each ROI and for each stimulation condition in a Table and associated statistical test results that you ran to conclude that the width was not increased across stimulation conditions.

 Again: the statistical results are not described for the frequency distributions (Figure 3 bottom panel). Please describe the statistical tests you ran to compare these across stimulation conditions and their outputs to arrive at your conclusion regarding substantial overlap. The distributions for GM and Pz do appear to overlap but they are non-overlapping for FC5 and FP2; the FP2 results are reported to have emerged from poorer image quality of the no-tDCS condition data, but what about for FC5?

**Discussion**

 It will help the reader if the authors are a bit more specific about their tDCS protocol in the second paragraph in the Discussion. The authors should mention the tDCS targets (cephalic, frontal anodal and cathodal tDCS electrode placement) and tDCS configurations (conventional and HD) that they assessed. These details are important to highlight that the procedures and safety measures, and image quality discussed in this paper are specific to conventional tDCS configurations and frontal montages, which would have to do be modified, say to be used with HD-tDCS configurations and for an anterior-posterior (e.g., frontal-parietal intra or interhemispheric) tDCS montage.  

**Minor:**

 First line in the Introduction: Consider replacing with “one of several methods of non-invasive …”.

 tDCS equipment and procedure: in point (1), replace . with ,

 Discussion: replace ‘tDCS behavioural effects’ with tDCS’ behavioural effects.

Is the rationale for developing the new method (or application) clearly explained?

Yes

Is the description of the method technically sound?

Yes

Are the conclusions about the method and its performance adequately supported by the findings presented in the article?

Yes

If any results are presented, are all the source data underlying the results available to ensure full reproducibility?

Yes

Are sufficient details provided to allow replication of the method development and its use by others?

Yes

Reviewer Expertise:

non-invasive brain stimulation, language, aphasia

I confirm that I have read this submission and believe that I have an appropriate level of expertise to confirm that it is of an acceptable scientific standard, however I have significant reservations, as outlined above.

## References

[1] : Transcranial Direct Current Stimulation as a Therapeutic Tool for Chronic Pain. *J ECT* .34(3) : 10.1097/YCT.0000000000000518 e36-e50 10.1097/YCT.0000000000000518 29952860PMC6105447
